# Skin development in the gray short‐tailed opossum (*Monodelphis domestica*)—From skin respiration to thermoregulation

**DOI:** 10.1111/joa.14236

**Published:** 2025-02-20

**Authors:** Kirsten Ferner

**Affiliations:** ^1^ Museum für Naturkunde Leibniz‐Institut für Evolutions‐ und Biodiversitätsforschung Berlin Germany

**Keywords:** cutaneous respiration, dermal ontogenesis, marsupials, skin development, thermoregulation

## Abstract

Marsupials are born at an early stage of development, and compared to eutherians, skin development is slow, and a functional change during skin ontogenesis occurs. The skin development in 36 gray short‐tailed opossums (*Monodelphis domestica*) has been examined using histological, morphometric, and μCT methods during postnatal development from neonate to adult. The aim of the study is to follow the structural and functional transition of the skin in this immature marsupial species. Additionally, the postnatal development of the external appearance and the cardiac and respiratory systems is looked at to assess skin development in relation to the general development. The skin of the newborn gray short‐tailed opossum is thin and undifferentiated (no hair follicles, no sebaceous and sweat glands). Numerous subepidermal capillaries allow for gaseous exchange via the skin. A dense cutaneous capillary net with a high capillary volume density (0.25 ± 0.04) is present at term, indicating significant cutaneous gas exchange in the neonate. The capillary volume density decreases markedly during the first postnatal week (0.08 ± 0.01). In the same time period, the skin diffusion barrier increases from 27 ± 4 to 87 ± 1 μm. From this age on, the skin development is characterized by thickening of the different cutaneous layers and beginning formation of hair follicles. First, hair covering the skin, sweat glands, and subcutaneous fat are observed by day 28, indicating the onset of thermoregulation. The total skin thickness in the gray short‐tailed opossum increases from 58 μm at birth to 726 μm by day 35, when the pelage is fully developed. The cardiac and respiratory systems are immature at birth. A fenestrated interatrial septum is present for the first 4 days, allowing skin respiration. Between day 4 and day 7, the lung enters the saccular stage of lung development and is mature enough to meet the respiratory needs of the growing organism. During a long period of postnatal development, the structural differentiation of the skin results in a functional shift from transcutaneous gas exchange to thermoregulation in later life.

## Graphical abstract

The skin development in the gray short‐tailed opossum (*Monodelphis domestica*) has been examined using histological, morphometric, and μCT methods. During a long period of postnatal development, the structural differentiation of the skin results in a functional shift from transcutaneous gas exchange to thermoregulation in later life.

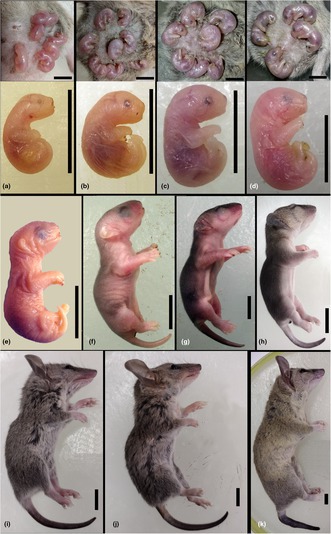

## 1 INTRODUCTION

In mammals, the skin serves several vital functions, including protection (physical barrier, UV radiation), regulation of body temperature (sweat production, vasodilation/‐constriction), sensation (sensory receptors), water balance (reduces evaporation), storage of fat (insulation, padding), vitamin D synthesis, and immunity. These functions make the skin essential for survival and allow mammals to adapt to varying environmental conditions.

Although these functions are similar in the skin of adult mammals, there are differences in the developmental rate and even a functional change during skin ontogenesis.

While cutaneous respiration, a form of gas exchange where oxygen and carbon dioxide diffuse directly through the skin bypassing the lungs, is a critical mode of respiration in many amphibians (e.g. Spearman, 1968), it is not a primary method for gas exchange in most mammals. However, limited forms of cutaneous respiration can occur during certain life stages, such as the early postnatal period in marsupials.

Newborn marsupials and newly hatched monotremes are very small and extremely immature. During this early stage of development, particularly when they are attached to the mother's teat as in marsupials or stay in an underground nest as in monotremes, their skin is much thinner and more permeable than in newborn eutherians. This means that a limited amount of gas exchange might occur through the skin, supplementing the developing respiratory system (Ferner, 2018).

Marsupial neonates are always highly altricial compared to eutherian neonates. Most of their growth and development occurs postnatally, supported by a prolonged lactation period (Ferner et al., 2017; Ferner & Mess, 2011; Renfree, 2006). Corresponding to the altricial –precocial spectrum of eutherians, marsupial neonates can also be sorted into three grades of developmental complexity (G1, G2, G3), based on size variation and the developmental degree of their organ systems (Hughes & Hall, 1988). Whereas dasyurid marsupials are the least developed (G1), peramelids, phalangerids, and didelphids are intermediately developed (G2) and macropodids and phascolarctids have the most developed marsupial neonates (G3).

The South American gray short‐tailed opossum (*Monodelphis domestica*) [Wagner, 1842] belongs to the family Didelphidae within the marsupial order Didelphimorphia. It is a small (80–120 g), carnivorous nocturnal marsupial native to Brazil and adjacent countries (VandeBerg & Robinson, 1997). It produces large litters (typically 6–13) after a gestation period of 13.5 days (Tyndale‐Biscoe & Renfree, 1987). The newborn is 8–9 mm long and weighs 100–120 mg (Szdzuy, 2006; Tyndale‐Biscoe & Renfree, 1987). The skeleton is cartilaginous, and the internal organs are visible through the transparent skin. The lungs are at the canalicular stage and consist of large terminal air spaces (Ferner, 2024a), and the kidneys are a functioning mesonephros (Ferner et al., 2017) at the time of birth.

The immaturity of the respiratory system of the newborn gray short‐tailed opossum suggests the recruitment of an alternative organ system such as the skin for gas exchange at least for a restricted time period, similarly to dasyurid marsupials (Frappell & MacFarlane, 2006; Frappell & Mortola, 2000; Ferner, 2018, 2021a).

Respiratory measurements in neonates of two dasyurid species, the fat‐tailed dunnart (*Sminthopsis crassicaudata*; Frappell & MacFarlane, 2006; Simpson et al., 2011) and Julia Creek dunnart (*Sminthopsis douglasi*; Frappell & Mortola, 2000), revealed no thoracic movement and pulmonary ventilation. In these most immature marsupials, gas exchange has been conducted solely via cutaneous respiration for a number of days, and the onset of pulmonary ventilation was severely delayed (Frappell & MacFarlane, 2006; Mortola et al., 1999). But even in comparatively well‐developed marsupial neonates, such as the tammar wallaby (*Macropus eugenii*), gas exchange through the skin accounts for 33% of the total oxygen uptake (MacFarlane et al., 2002; MacFarlane & Frappell, 2001). The developmental degree of the newborn gray short‐tailed opossum is somewhere in between, and the rate of cutaneous respiration might be around 50%–60% (Ferner, 2018).

Earlier morphological studies of skin development in marsupials neglected parameters important for cutaneous respiration, such as capillary density or air‐blood diffusion distances (Armstrong & Ferguson, 1995; Krause et al., 1978; Lyne, 1970; Lyne et al., 1970; Pralomkarn et al., 1990). Studies on gas transfer in 3‐day‐old pouch young tammar wallabies (*M. eugenii*, 370 mg) largely dismissed the possibility that the skin may be an accessory or a major site of gas exchange (Baudinette et al., 1988; Randall et al., 1984). Recent morphological and morphometric studies on skin development in several marsupial species reported a thin blood‐air diffusion barrier and high subepidermal capillarization during the first postnatal days, suggesting that skin gas exchange might play a role in the early postnatal period in marsupials (Ferner, 2018, 2021a; Makanya et al., 2007).

However, a thin epidermis to facilitate gas exchange via the skin threatens the marsupial offspring by desiccation. Marsupials are generally considered to be raised in a pouch; in those species that do, the pouch offers a thermally stable and humid environment in which to raise the young (Frappell & MacFarlane, 2006). However, not all species have the well‐developed pouches characteristic of wallabies and kangaroos (macropodids). The gray short‐tailed opossum is a pouch‐less species, and the young have to cope with the outside environment. There seems to be a tradeoff to allow for permeability necessary for efficient cutaneous gas exchange on one side and to avoid excessive water and heat loss via the skin on the other side. Over time, other functions of the skin (protection, insulation, reducing water loss) become more important, and structural changes have to take place to address these issues.

Whereas protection and reduction of water loss can be addressed by thickening of the epidermis, insulation and attaining thermoregulation are tightly linked to the development of pelage. In marsupials, initially the thermogenic response is small, but gradually the maximal thermogenesis increases until it is similar to that of the adult (Geiser et al., 1986; Gemmell & Cepon, 1993; Setchell, 1974). During this period of gradual attainment of thermogenic capacity, hair growth begins, and by the time full thermogenic capacity is reached, a complete pelage is present. Full thermogenic capacity coincides with the attainment of the adult metabolic level (Szdzuy & Zeller, 2009).

All marsupial young are born without hair and are incapable of maintaining a steady rectal temperature. The thyroid gland, an endocrine gland that influences skin structure and thermoregulation, is not present in the newborn marsupial. Thyroid function, production of hair, and thermogenic capacity in the marsupial do not occur until approximately two‐thirds of the way through pouch occupancy (Hulbert, 1988; Pralomkarn et al., 1990). These activities occur in the rat and mouse just before or shortly after birth and in the fetal sheep during late gestation (Johnston & Gemmell, 1987). The appearance of hair and sebaceous and sweat glands at day 67 postpartum in the marsupial native cat supports the view that thermogenic capacity is developed in the latter part of pouch life (Pralomkarn et al., 1990).

The nature and the site of the endothermic thermogenesis are not clear, but there may be both shivering and non‐shivering components (Hulbert, 1988). Adaptive non‐shivering thermogenesis generated in brown adipose tissue (BAT) enables small eutherian mammals, such as rodents, newborns, and hibernating mammals, to maintain high body temperature independent of daily and seasonal temperature fluctuations (Cannon & Nedergaard, 2004). The successful radiation of eutherian mammals to cold environments was most likely facilitated by classical adaptive non‐shivering thermogenesis depending on BAT and its crucial uncoupling protein UCP1 (Cannon & Nedergaard, 2004). Evidence for non‐shivering thermogenesis is a matter of debate in marsupial mammals, which separated from eutherians about 150 million years ago (Bininda‐Emonds et al., 2007). In some marsupials like *M. domestica* or *Antechinus flavipes*, UCP1 may be recruited transiently during early stages of development and is lost during adulthood (Jastroch et al., 2007).

The current study uses histological, morphometric, and Micro‐CT techniques to investigate the structural development of the skin of *M. domestica* during the postnatal period, addressing a functional transformation from cutaneous respiration to thermoregulation. In addition, external appearance and cardiac and pulmonary development are included as reference data to assess skin development in relation to the general development.

## 2 MATERIALS AND METHODS

### 2.1 Animals

**FIGURE 1:**
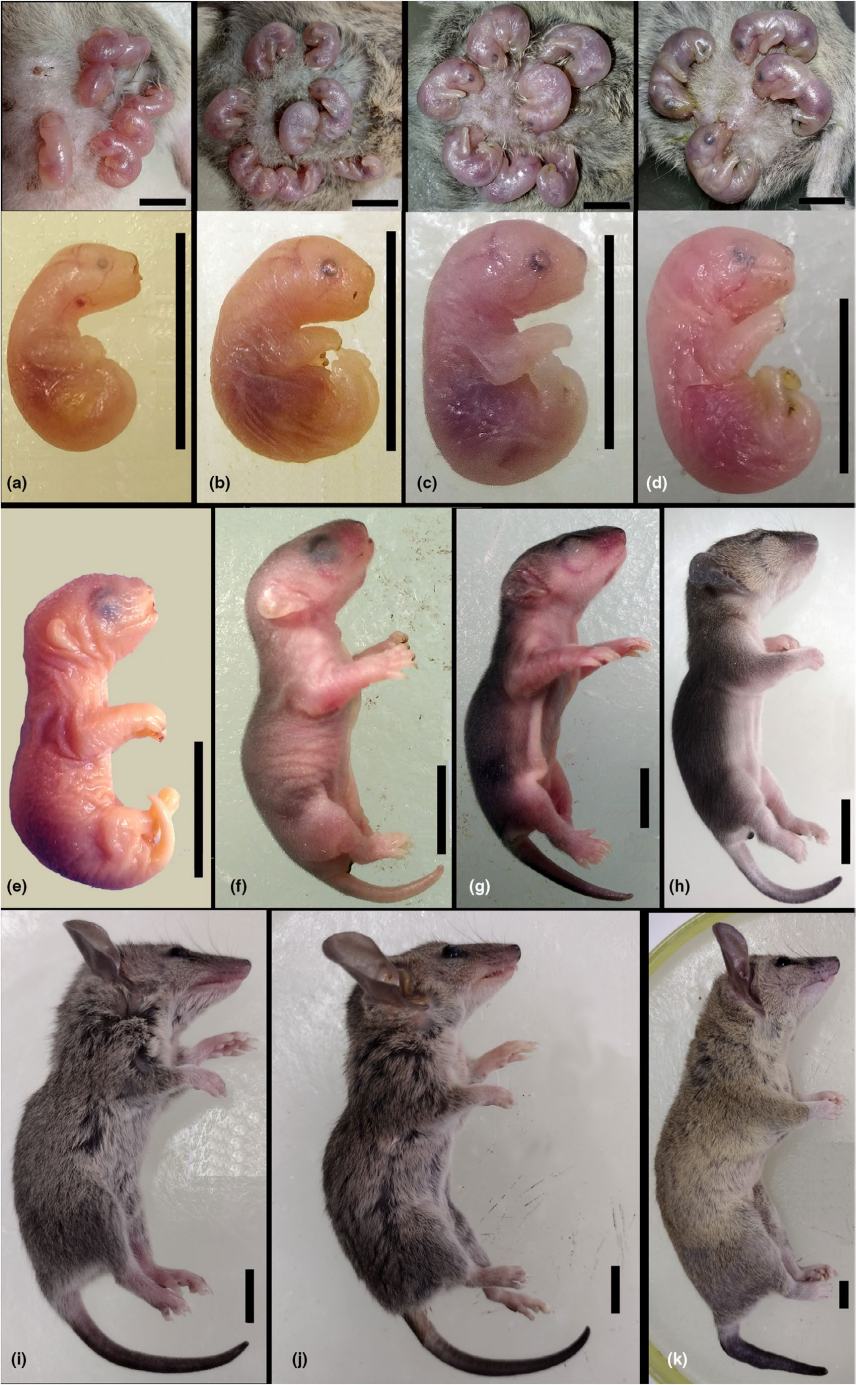
Postnatal development of *Monodelphis domestica*. Macrographs reflect the morphological transformation from neonate to adult: neonate (a), 4 dpn (b), 7 dpn (c), 11 dpn (d), 14 dpn (e), 21 dpn (f), 28 dpn (g), 35 dpn (h), 49 dpn (i), 57 dpn (j), adult (k). The marsupial young are firmly attached to the maternal teat immediately after birth and remain permanently attached until day 11 (see top panel). The highly immature neonate has strongly developed forelimbs and rudimentary hind limbs, an oral shield, and large prominent nostrils. The eye primordium shows retinal pigmentation, and ear primordia are not visible. During the first week, the body posture is still curled up. Around day 11, the hind limbs start to differentiate, and digits become recognizable. A clear developmental progress was observed between day 14 and day 21, where the area of the later mystacial vibrissae and eyelids, and auricle become visible but were still fused. The oral fissure is formed, and the young are not permanently attached anymore. The morphological transformation of forelimbs and hindlimbs to their final posture progresses. Pinnae became free from the head around day 28; eyes and ears are open now. First hairs erupt on the head around day 21, reaching the middle back by day 28. The young are completely furred by day 35. During the late postnatal period, the young become larger, and except for the proportionally bigger heads, they resemble the adult animals in external appearance. Scale bar = 1 cm. Details of the specimens are provided in Table 1. dpn, day post natum.

Skin development was examined in 36 gray short‐tailed opossums (*M. domestica*), which were part of a study on lung development. The females were controlled mated for this study and checked for offspring when approaching full‐term (13–14 days). Young ranging from birth to 57 days post natum (dpn) and adults (primi‐ or multiparous females 1 year old) were collected, weighed, and euthanized by anesthetic overdose with isoflurane under an animal ethics permit approved by the Animal Experimentation Ethics Committee (registration number: T0202/18). To assess possible changes around parturition, one female was euthanized shortly before term by day 13 of gestation, and the embryos were dissected and fixed by Karnovsky's fixative (Mulisch & Welsch, 2015). The numbers and specifics of the specimens used in this study are summarized in a table (Supplement). The term neonate is defined as the first 24 at the day of birth. The young remain permanently attached to the teats for 14 days, open their eyes between 28 and 35 days, leave the nest around 35 days, and are weaned by 49 days (Szdzuy, 2006). The morphological development from neonate to adult is presented in Figure 1.

### 2.2 Sample preparation for light microscopy

Early developmental stages, ranging from neonate to 21 dpn, were decapitated to allow for better fixation results. The whole body of the animals was immediately immersed in Karnovsky's fixative (2 g paraformaldehyde, 25 mL distilled water, 10 mL 25% glutaraldehyde, 15 mL 0.2 M phosphate buffer; Mulisch & Welsch, 2015). In later developmental stages, from 28 dpn to adults, a part of the dorsal skin (epidermal and dermal layers) was dissected and fixed by Karnovsky's fixative. The fixation time was usually 1–2 days. Afterwards, the specimens were rinsed in 70% ethanol and embedded in paraffin (Shanon Hypercenter XP, Life Science International GmbH, Frankfurt, Germany). The specimens were serially sectioned in a transverse plane. The sections (thickness 4 μm) were stained with Azan, trichrome, or hematoxylin and eosin.

### 2.3 μCT imaging

Comparative, functional, and developmental studies of animal morphology require accurate visualization of three‐dimensional structures. μCT imaging offers the possibility of nondestructive whole‐volume imaging of animal tissues and is appropriate to study the subcutaneous capillary network and the interatrial septum of the heart. For the examination of lung development, torsos of 13 dpc (days post coitum), neonate, 2, and 4 dpn were stained with 1% phosphotungstic acid (PTA) and subjected to μCT analysis at the Museum für Naturkunde Berlin (lab reference ID SCR_022585) using a Phoenix nanotom X‐ray machine (Waygate Technologies, Baker Hughes, Wunstorf, Germany; equipment reference ID SCR_022582). Details for staining and scanning of the specimen used in this study were described previously in detail (Ferner, 2024a, 2024b; Ferner & Mahlow, 2023). The 3D volume processing of the subcutaneous capillary layer as well as of the interatrial septum of the heart was carried out with the software Volume Graphics Studio Max Version 3.5 (Volume Graphics GmbH, Heidelberg, Germany). μCT data were analyzed as serial two‐dimensional (2D) and reconstructed to three‐dimensional (3D) images (Figure 3). A region grower tool was used that marks all areas of the same density value connected to each other to create a region of interest (ROI). For details of the segmentation process, see previous studies (Ferner, 2024a, 2024b; Ferner & Mahlow, 2023).

### 2.4 Morphometric measurements

Morphometric measurements of the skin were the subject of former morphological studies and described in detail elsewhere (Ferner, 2018, 2021a).

**FIGURE 2:**
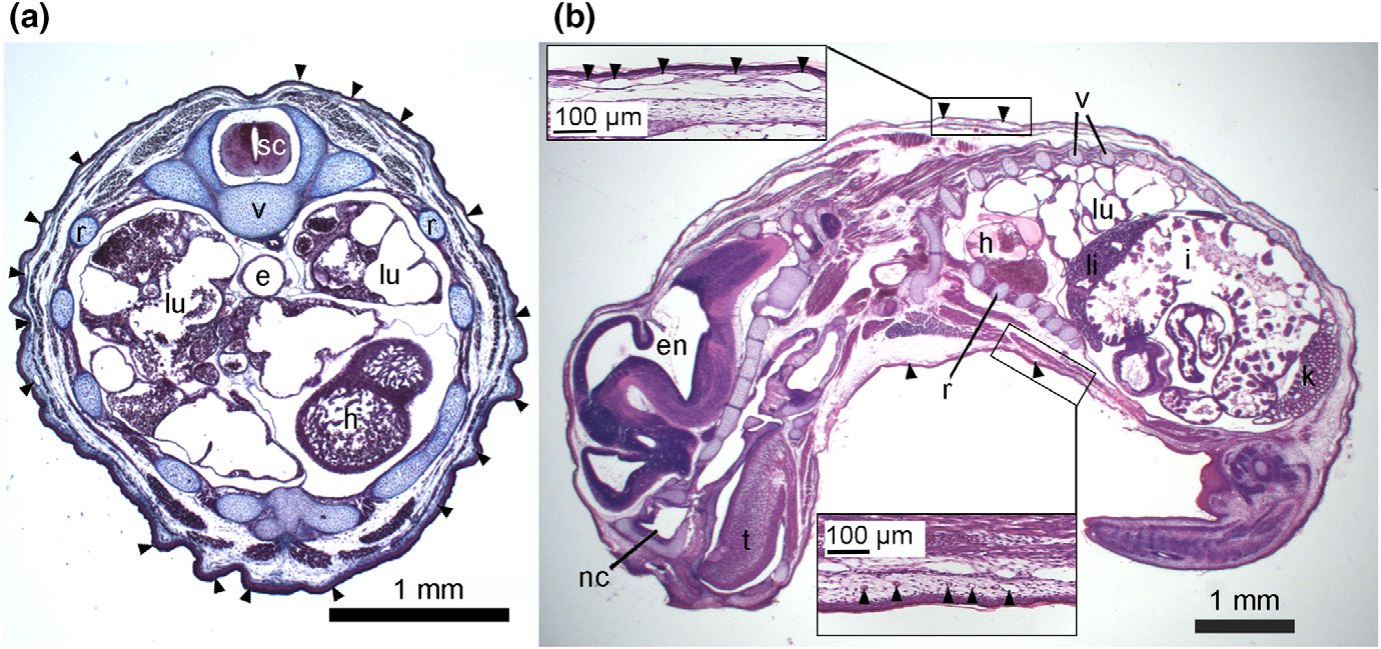
Light micrographs of a transversal (a) and a longitudinal (b) histological section through a newborn *Monodelphis domestica*. Numerous superficial capillaries (arrowheads) are closely associated with the epidermis, providing an extremely thin diffusion barrier. Cutaneous capillaries are present in the dorsal, lateral, and ventral sides; however, capillary density is highest in the skin of the dorsolateral side of the trunk (b, insets). The general developmental degree of *M. domestica* is highly immature at birth, showing cartilaginous skeletal elements, lungs at the canalicular period, and poor development of the gut and associated organs (i.e., liver, intestine, mesonephric kidney). e, esophagus; en, encephalon; h, heart; i, intestine; k, kidney; lu, lung; m, musculature; nc, nasal cavity; r, rib; sc, spinal cord; t, tongue; v, vertebrae.

Subcutaneous capillaries were present in the skin of the ventral, dorsal, and lateral sides of the trunk (Figure 2). A preceding study suggested a higher volume density of cutaneous capillaries in the dorsolateral side of the trunk (Vvc = 0.33 ± 0.05), whereas the capillary volume density was lower in the ventral side of the trunk (Vvc = 0.14 ± 0.02) (Ferner, 2018). Considering the lack of a pouch, the curled‐up position of the neonates, and the close proximity to litter mates, the dorsolateral side of the trunk is the only body region freely exposed to the air and seems to be the most important site for cutaneous gas exchange. Therefore, the measurements of the skin were conducted on the dorsolateral side of the torsos in early‐stage animals and the dorsal skin samples of older animals.

Serial histological sections were investigated by light microscopy using a stereomicroscope (Leica MZ 12; Wildt, Switzerland), and morphometric methods were applied (Figure 4). To ensure that selected tissue samples represent the whole, all parts of the dorsolateral side of the trunk should have equal probability of being sampled (Hsia et al., 2010). This requirement is met by introducing randomness into the sampling process. For this reason, the Fractionator method was applied. This is a comprehensive sampling approach that is based on serial sectioning through the entire specimen, followed by systematic selection of a known fraction of the whole. Since the section thickness (usually 4 μm) was known, the total number of sections resulted in the total length of the specimen. The total length divided by 10 gave the sampling thickness for the fractionator. Ten sections per specimen were selected for measurement. In order to choose arbitrary positions for measurement on the dorsolateral side of the trunk, an orientator clock was applied (Howard & Reed, 2005). As a result, 10 selected skin sections of each specimen were captured with a magnification of 200× (13 dpc‐14 dpn) or 100× (21 dpn‐adult) on a light microscope (Zeiss Axiokop, Carl Zeiss Microscopy GmbH, Germany) equipped with a digital camera (Leica DFC490, Leica Microsystems, Switzerland Ltd.), connected to a computer (software LAS V4.2, Leica Microsystems, Switzerland Ltd.). Assessment of total skin thickness and of its different components (dermis, epidermis, stratum corneum, periderm) as well as the distance between the external surface and most superficial capillaries was performed at 5 different points on the image (Fractionator principle), yielding 50 measurements per specimen. Measurements were made directly on the computer screen using a digital ruler (Image J software, National Institutes of Health, USA). The results of the measured slides have been averaged for each animal, and the mean and SE were presented. Additional group means for the age stage (bold) are given (Table 1).

**TABLE 1:** Cutaneous morphometric data of *Monodelphis domestica* during the postnatal period.

Age	Animal No.	Entire skin thickness, μm	Dermal thickness, μm		Epidermal thickness, μm	Stratum corneum thickness, μm	Peridem thickness, μm	Diffusion distance, μm	Volume density of capillaries in the skin, Vvc	Volume density of hair follicles in the skin, Vvh	Volume density of sweat glands in the skin, Vvs
			Papillar	Reticular							
13 dpc	2095e	54 ± 2	41 ± 1	‐	8 ± 0	2 ± 1	2 ± 0	23 ± 1	0.39 ± 0.03	‐	
	2095g	63 ± 2	45 ± 1	‐	14 ± 0	3 ± 0	2 ± 0	26 ± 1	0.50 ± 0.04	‐	
	Mean	**58 ± 4**	**43 ± 2**	‐	**11 ± 3**	**3 ± 1**	**2 ± 0**	**24 ± 2**	**0.44 ± 0.06**	‐	
Neonate	337	64 ± 2	42 ± 2	‐	16 ± 0	5 ± 0	2 ± 0	39 ± 5	0.19 ± 0.02	‐	
	1965_1	51 ± 2	37 ± 2	‐	10 ± 0	2 ± 0	1 ± 0	26 ± 1	0.16 ± 0.01	‐	
	1965_5	65 ± 3	49 ± 3	‐	10 ± 0	4 ± 0	2 ± 0	23 ± 1	0.31 ± 0.02	‐	
	1965_6	52 ± 2	37 ± 2	‐	9 ± 0	4 ± 0	2 ± 0	21 ± 1	0.34 ± 0.05	‐	
	**Mean**	**58 ± 4**	**41 ± 3**	‐	**11 ± 2**	**3 ± 0**	**2 ± 0**	**27 ± 4**	**0.25 ± 0.04**	‐	
2 dpn	2381_1	59 ± 2	41 ± 2	‐	11 ± 0	4 ± 0	3 ± 0	35 ± 2	0.26 ± 0.02	‐	
	2381_2	60 ± 3	44 ± 2	‐	11 ± 0	4 ± 0	2 ± 0	31 ± 1	0.31 ± 0.03	‐	
	2381_3	69 ± 2	51 ± 2	‐	15 ± 0	4 ± 0	2 ± 0	40 ± 1	0.24 ± 0.02	‐	
	**Mean**	**62 ± 3**	**45 ± 3**	‐	**12 ± 2**	**3 ± 0**	**2 ± 0**	**35 ± 2**	**0.27 ± 0.02**	‐	
4 dpn	3003A	118 ± 5	86 ± 5	‐	25 ± 1	5 ± 0	2 ± 0	73 ± 4	0.14 ± 0.00	‐	
	1995_2	111 ± 5	88 ± 5	‐	20 ± 1	2 ± 0	1 ± 0	76 ± 4	0.07 ± 0.01	‐	
	1825	92 ± 2	63 ± 2	‐	23 ± 1	4 ± 0	2 ± 0	58 ± 2	0.12 ± 0.01	‐	
	**Mean**	**107 ± 8**	**79 ± 8**	‐	**23 ± 2**	**4 ± 1**	**2 ± 1**	**69 ± 5**	**0.11 ± 0.02**	‐	
7 dpn	3006A	121 ± 3	95 ± 3	‐	19 ± 1	6 ± 0	‐	86 ± 3	0.07 ± 0.00	0.09 ± 0.02	
	2383_2	125 ± 5	98 ± 5	‐	20 ± 1	7 ± 0	‐	88 ± 4	0.10 ± 0.03	0.03 ± 0.01	
	2383_4	106 ± 4	77 ± 3	‐	25 ± 1	4 ± 0	‐	89 ± 4	0.08 ± 0.01	0.06 ± 0.01	
	**Mean**	**117 ± 6**	**90 ± 7**	‐	**21 ± 2**	**6 ± 1**	‐	**87 ± 1**	**0.08 ± 0.01**	**0.06 ± 0.02**	
11 dpn	2419_1	195 ± 5	115 ± 6	43 ± 3	38 ± 1	7 ± 0	‐	152 ± 7	0.05 ± 0.01	0.08 ± 0.01	
	2419_2	159 ± 4	96 ± 4	31 ± 1	39 ± 1	4 ± 0	‐	112 ± 4	0.04 ± 0.00	0.13 ± 0.01	
	2419_7	179 ± 11	90 ± 3	59 ± 11	23 ± 1	9 ± 0	‐	128 ± 3	0.04 ± 0.00	0.09 ± 0.00	
	**Mean**	**178 ± 1**	**101 ± 7**	**44 ± 8**	**34 ± 5**	**7 ± 2**	‐	**131 ± 12**	**0.04 ± 0.00**	**0.10 ± 0.01**	
14 dpn	3014A	249 ± 4	126 ± 5	100 ± 2	18 ± 1	5 ± 0	‐	191 ± 8	0.03 ± 0.00	0.14 ± 0.01	
	1994_8	234 ± 4	101 ± 5	109 ± 3	17 ± 1	7 ± 0	‐	218 ± 6	0.02 ± 0.01	0.16 ± 0.01	
	1994_9	245 ± 5	106 ± 4	114 ± 3	20 ± 1	5 ± 0	‐	205 ± 5	0.03 ± 0.01	0.16 ± 0.01	
	**Mean**	**243 ± 4**	**111 ± 8**	**108** ± 4	**18 ± 1**	**6 ± 1**	‐	**205 ± 8**	**0.03 ± 0.00**	**0.16 ± 0.01**	
21 dpn	2038	459 ± 4	121 ± 5	312 ± 2	20 ± 1	6 ± 0	‐	365 ± 8	0.02 ± 0.00	0.35 ± 0.01	
	2039	480 ± 4	125 ± 5	328 ± 3	21 ± 1	6 ± 0	‐	412 ± 6	0.02 ± 0.01	0.38 ± 0.01	
	**Mean**	**469 ± 9**	**123 ± 1**	**320** ± 7	**21 ± 1**	**6 ± 0**	‐	**389 ± 20**	**0.02 ± 0.00**	**0.37 ± 0.01**	
28 dpn	2041	641 ± 10	207 ± 7	414 ± 8	15 ± 1	5 ± 1	‐	586 ± 9	0.01 ± 0.00	0.36 ± 0.01	0.012 ± 0.001
	2042	590 ± 14	167 ± 5	406 ± 12	12 ± 0	5 ± 0	‐	512 ± 14	0.01 ± 0.00	0.34 ± 0.01	0.004 ± 0.000
	2043	599 ± 13	192 ± 5	388 ± 9	14 ± 0	5 ± 0	‐	554 ± 15	0.01 ± 0.00	0.38 ± 0.00	0.007 ± 0.003
	**Mean**	**610 ± 16**	**189 ± 12**	**403** ± 7	**14 ± 1**	**5 ± 0**	‐	**551 ± 21**	**0.01 ± 0.00**	**0.36 ± 0.01**	**0.008. ± 0.002**
35 dpn	2061	730 ± 20	186 ± 6	516 ± 15	21 ± 1	7 ± 0	‐	640 ± 22	0.02 ± 0.00	0.41 ± 0.01	0.009 ± 0.001
	2062	706 ± 25	146 ± 7	574 ± 22	10 ± 0	5 ± 0	‐	667 ± 29	0.01 ± 0.00	0.42 ± 0.01	0.009 ± 0.002
	2065	742 ± 16	215 ± 5	505 ± 16	17 ± 0	5 ± 0	‐	688 ± 9	0.01 ± 0.00	0.40 ± 0.01	0.009 ± 0.002
	**Mean**	**726 ± 11**	**182 ± 20**	**532** ± 217	**16 ± 3**	**6 ± 1**	‐	**665 ± 14**	**0.01 ± 0.00**	**0.41 ± 0.01**	**0.009. ± 0.000**
49 dpn	2045	598 ± 7	175 ± 4	404 ± 6	14 ± 0	5 ± 0	‐	485 ± 11	0.01 ± 0.00	0.35 ± 0.01	0.013 ± 0.002
	2046	600 ± 20	197 ± 8	381 ± 16	16 ± 1	5 ± 0	‐	512 ± 19	0.02 ± 0.00	0.28 ± 0.00	0.013 ± 0.002
	2402	564 ± 7	199 ± 6	345 ± 6	15 ± 0	5 ± 0	‐	463 ± 7	0.01 ± 0.00	0.28 ± 0.01	0.007 ± 0.001
	**Mean**	**587 ± 12**	**190 ± 8**	**377** ± 17	**15 ± 1**	**5 ± 0**	‐	**487 ± 14**	**0.01 ± 0.00**	**0.30 ± 0.02**	**0.011. ± 0.002**
57 dpn	1642	471 ± 4	257 ± 3	198 ± 4	12 ± 1	5 ± 0	‐	418 ± 7	0.01 ± 0.00	0.22 ± 0.01	0.015 ± 0.003
	2362_2	420 ± 28	219 ± 15	181 ± 7	15 ± 1	6 ± 0	‐	340 ± 13	0.01 ± 0.00	0.19 ± 0.01	0.021 ± 0.002
	**Mean**	**446 ± 26**	**238 ± 19**	**189 ± 9**	**13 ± 1**	**5 ± 0**	‐	**379 ± 39**	**0.01 ± 0.00**	**0.20 ± 0.02**	**0.018. ± 0.003**
Adult	1986	547 ± 12	308 ± 7	222 ± 9	12 ± 0	6 ± 0	‐	478 ± 10	0.01 ± 0.00	0.16 ± 0.02	0.001 ± 0.001
	2381	657 ± 11	243 ± 5	396 ± 9	12 ± 0	6 ± 0	‐	549 ± 10	0.01 ± 0.00	0.21 ± 0.01	0.009 ± 0.002
	2383	660 ± 13	433 ± 11	204 ± 8	18 ± 1	7 ± 0	‐	582 ± 9	0.02 ± 0.00	0.11 ± 0.01	0.015 ± 0.003
	**Mean**	**621 ± 37**	**328 ± 56**	**274** ± 61	**14 ± 2**	**6 ± 0**	‐	**536 ± 31**	**0.01 ± 0.00**	**0.16 ± 0.03**	**0.008 ± 0.004**

*Note*: All values are means ± SE. For age stages with more than one specimen investigated, additional group means are presented.

Using LM morphometry, the volume densities of capillaries (Vvc), hair follicles (Vvh), and sweat glands (Vvs) in the 10 selected skin sections were obtained by point‐counting methods (Howard & Reed, 2005), following the equation:

Vvc=Pc/Ptot;Vvh=Ph/Ptot;Vvs=Ps/Ptot

where Pc, Ph, Ps, and Ptot are point counts on capillaries, hair follicles, sweat glands, and the total number of points on the skin tissue. The sample size per specimen was 200 point counts on each of the 10 selected skin images. The resulting 10 volume densities were averaged for each animal, the mean and SE were presented, and again group means for age stages were presented (Table 1).

## 3 RESULTS

### 3.1 General morphological development

Body size and external morphology of the developing young gray short‐tailed opossum provide important information for evaluating properties for cutaneous gas exchange and thermoregulation. The morphological development from neonate to adult is presented in Figure 1 and body weight is given in Table 1.

The crown–rump length (CRL) of the newborn *M. domestica* is 8.5 mm, and body weight is about 0.11 g. The body posture of the newborn *M. domestica* is curled up to the ventral side, resulting in an embryonic appearance (Figure 1a). The neonate has strongly developed muscular forelimbs with claws and undifferentiated hindlimb paddles. The medial nasal swelling contributes to a large prominent nostril. After the newborn is attached to the maternal teat, the oral fissures merge and form an oral shield, ensuring that the young stay firmly attached to the mother. The eye primordium shows retinal pigmentation, but the ear primordium is not discernible. The skin appears translucent, and internal organs and capillaries are visible through the skin. By day 4, the crown–rump length (CRL) is 11 mm, and the body weight is 0.2 g. The body posture is still curled up (Figure 1b). Eyes and ears are closed, with no auricles discernible. The phalanges of the fore and hind limbs develop. By day 7, the CRL measures 13 mm, and body weight is about 0.3 g. The body is stretching, and the spine becomes straight (Figure 1c). The auricles are indicated by a fold, but ears and eyes are still closed. By day 11, the CRL measures 15 mm, and the body weight is 0.4 g (Figure 1d). The differentiation of the hind limbs progresses, and by day 11, the phalanges are clawed. The fore and hind limbs are at the same developmental stage now. The later mouth opening and auricles are indicated by corresponding folds. By day 14, the CRL is 20 mm, and the body weight is 1 g (Figure 1e). The oral fissure is formed, and the young are not permanently attached anymore. The facial development progresses rapidly by day 14. Ear and eye primordia, the palpebral fissures, and the area of the later mystacial vibrissae become visible. The morphological transformation of fore‐ and hindlimbs progresses by this time. By day 21, the CRL is 38 mm, and the body weight is about 2.4 g (Figure 1f). The eyes are still closed. There is an increasingly darker pigmentation on the back at this time. The young remain often in a nest and are left by the mother for a short time. By day 28, the CRL measures 45 mm, and body weight is about 4.6 g (Figure 1g). Pinnae were free from the head around day 28; eyes and ears are open now. First hairs appear on the head and the middle back, resulting in a darker coloration of these body parts. By day 35, the CRL is 50 mm, and the body weight is about 7 g (Figure 1h). By this time, the young are completely furred. The young are able to coordinate locomotion and leave the nest occasionally. The young start eating solid food. By day 49 and 57, the CRL is 65 and 75 mm, and the body weight is about 12 and 18.5 g, respectively (Figure 1i,j). The young gain in weight and become larger. Except for the proportionally bigger heads, they resemble the adult animals in external appearance. Between day 49 and 57, the young are weaned and become independent. The animals reach sexual maturity around 180 days. Adult animals have a CRL of 130 mm, and the body weight ranges from 60 to 100 g (Figure 1k).

### 3.2 Postnatal development of the skin

**FIGURE 3:**
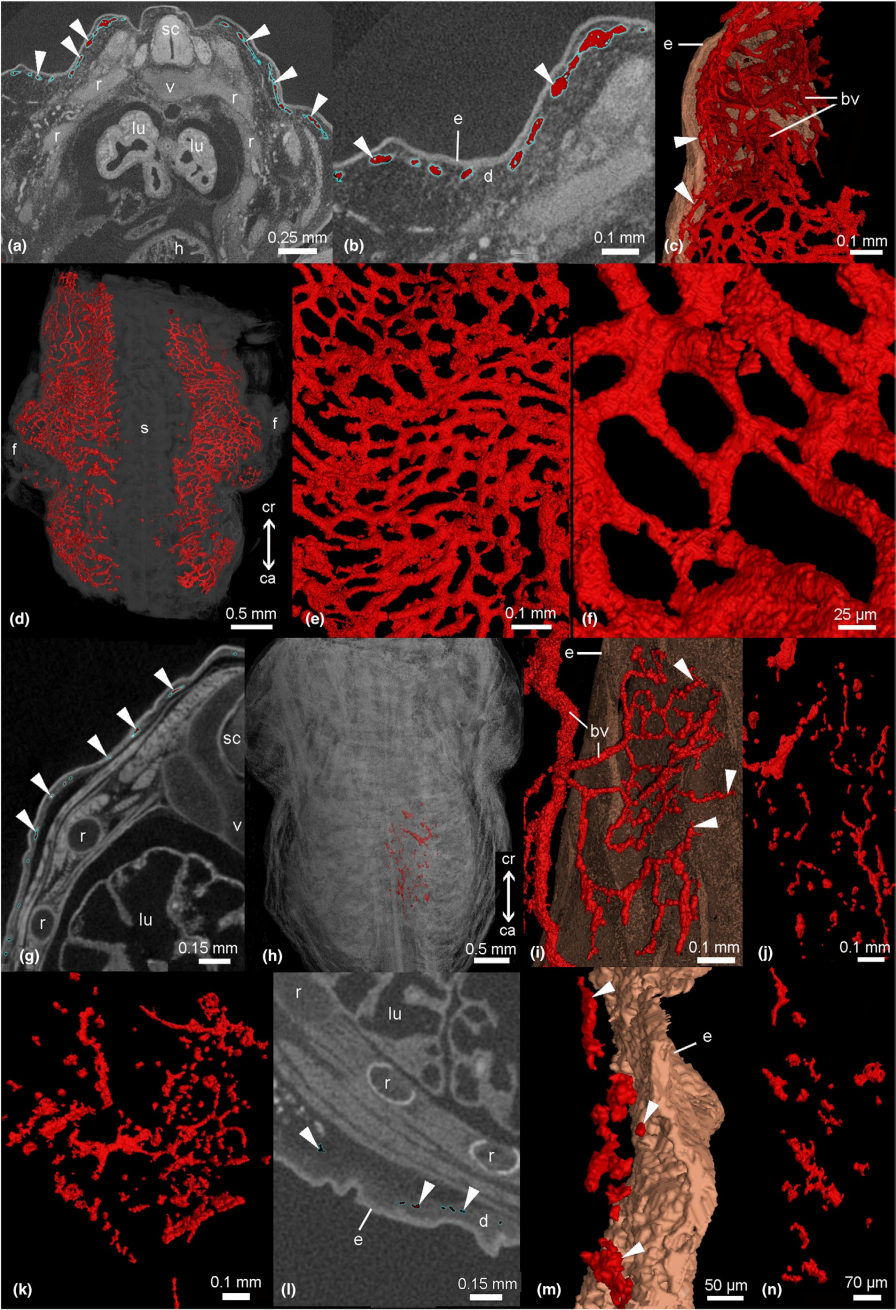
μCT images and 3D reconstructions of the cutaneous capillaries in the skin of the gray short‐tailed opossum by near term (a–f; 13 dpc), in the neonate (g–j), and by 2 dpn (k) and 4 dpn (l–n). The skin in the near‐term embryo has numerous subepidermal capillaries (a–b, indicated by arrowhead). 3D reconstruction of μCT scans of the skin reveals a dense cutaneous capillary net all over the body (d–f). In the newborn, capillaries are still numerous and densely connected, but they do not appear as a compact capillary net anymore (g–j). The subepidermal capillaries communicate with larger transport vessels, which join into larger blood vessels that communicate with the arterial and venous system (i). By day 2, cutaneous capillaries appear still dense in the dermis (k). By day 4, cutaneous capillaries can be seen less often; the capillary volume density of the skin decreases, and the diffusion distance increases due to dermal growth (l–n). bv, blood vessel; d, dermis; dpc, days post coitum; dpn, day post natum; epidermis; f, forelimb; h, heart; lu, lung; r, rib; s, spine; sc, spinal cord; v, vertebrae.

The skin at the dorsolateral side of the near‐term embryo and newborn gray short‐tailed opossum measures 58 ± 4 μm. It is composed of a thin layer of epidermis (11 ± 0 μm), which is in proximity to the capillary bed located on a layer of dermis (Figure 5a,b). The epidermis consists of one to two layers of squamous cells, which are followed by two layers of flattened partially keratinized cells, the stratum corneum (3 ± 0 μm, darker staining) and the outermost surface layer, the periderm (2 ± 0 μm, lighter staining; Figure 5b, right side with higher magnification). The dermis measures 41 ± 3 μm and consists of connective tissue and embedded capillaries. Hairs and hair follicles, sebaceous and perspiratory glands are completely missing in the skin of the newborn gray short‐tailed opossum. Numerous superficial capillaries are encountered close to the epidermis (Figure 5a,b). Although cutaneous capillaries are present at the ventral side of the trunk as well (Figure 2), the capillary density is higher and the diffusion distance of subepidermal capillaries lower on the dorsolateral side of the trunk. The capillary volume density of the skin is highest in the near‐term embryo (0.44 ± 0.06). 3D reconstruction of μCT scans reveals a dense cutaneous capillary net all over the body in the near‐term embryo (Figure 3a–f). In the newborn gray short‐tailed opossum, the capillary volume density of the skin is 0.25 ± 0.04 slightly decreased. Capillaries are still numerous and densely connected, but they do not appear as a capillary net anymore (Figure 3g–j). The distance between the external surface of the skin and the most superficial capillaries (skin diffusion barrier) in the dorsolateral skin of the newborn gray short‐tailed opossum measures 27 ± 4 μm.

**FIGURE 4:**
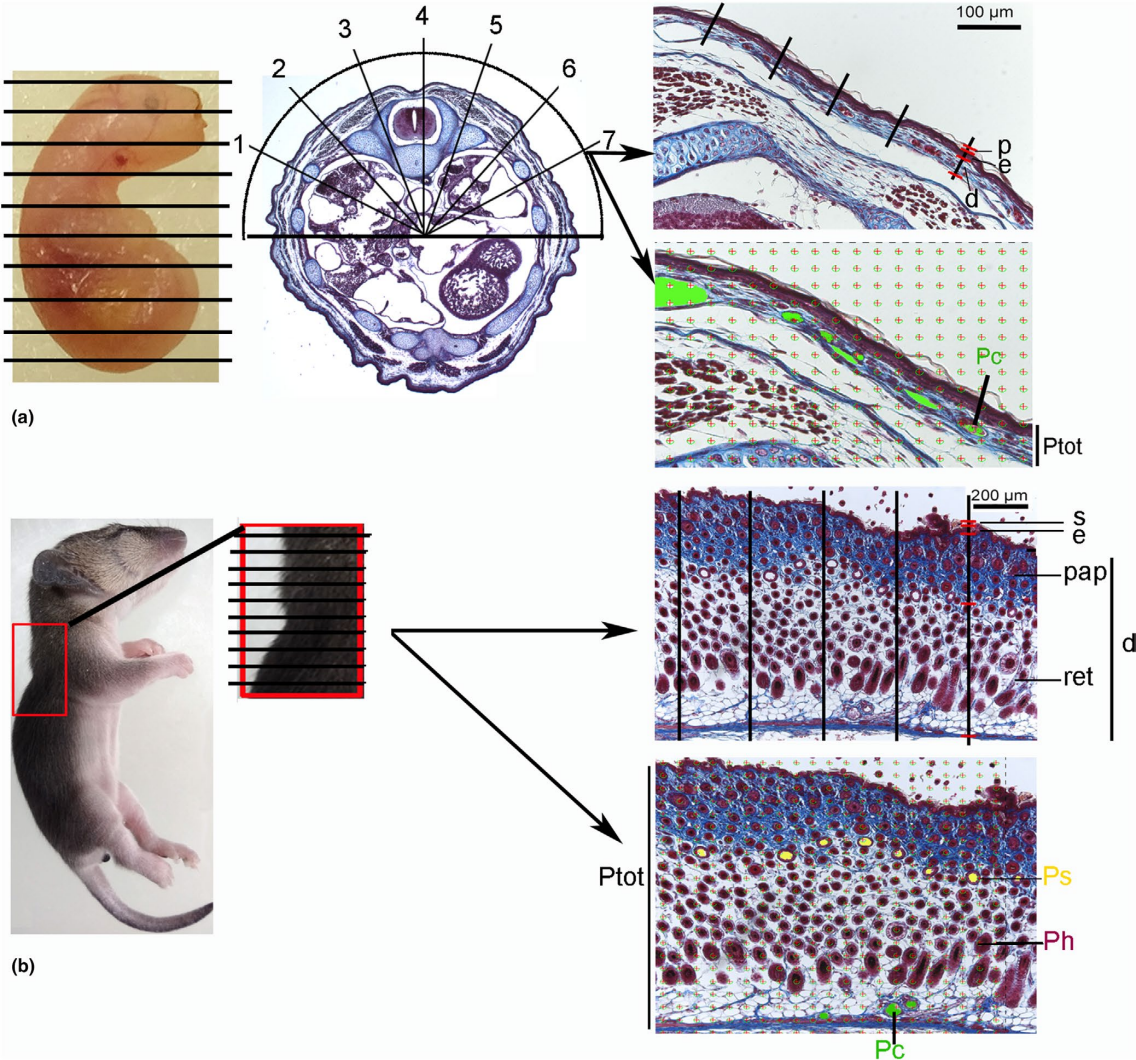
Morphometric methods applied for analyzing skin development in the gray short‐tailed opossum. First, the entire specimen (a, neonate‐21 days) or the dorsal skin samples (b, 35 days‐adult) were serial sectioned, followed by systematic selection of a known fraction of the whole (fractionator). The total length of the specimen or dorsal skin sample divided by 10 gave the sampling thickness for the fractionator. In the small specimen, an orientator clock was applied to choose random sections of the dorsolateral side of the torso. As a result, 10 selected skin sections of each specimen were captured with a magnification of 200× (torso) or 100× (skin) on a light microscope equipped with a digital camera. Measurement of total skin thickness and of its different components (dermis, epidermis, stratum corneum, periderm) as well as the diffusion distance was performed at 5 different points on the image (fractionator principle), yielding 50 measurements per specimen. Using morphometric point‐counting methods, the volume densities of capillaries (Vvc), hair follicles (Vvh), and sweat glands (Vvs) were obtained in the 10 selected skin sections per specimen. d, dermis; e, epidermis; p, periderm; pap, papillary dermis; Pc, points on capillaries; Ph, points on hair follicles; Ps, points on sweat glands; Ptot, total points on skin tissue; ret., reticular dermis; s, stratum corneum.

**FIGURE 5:**
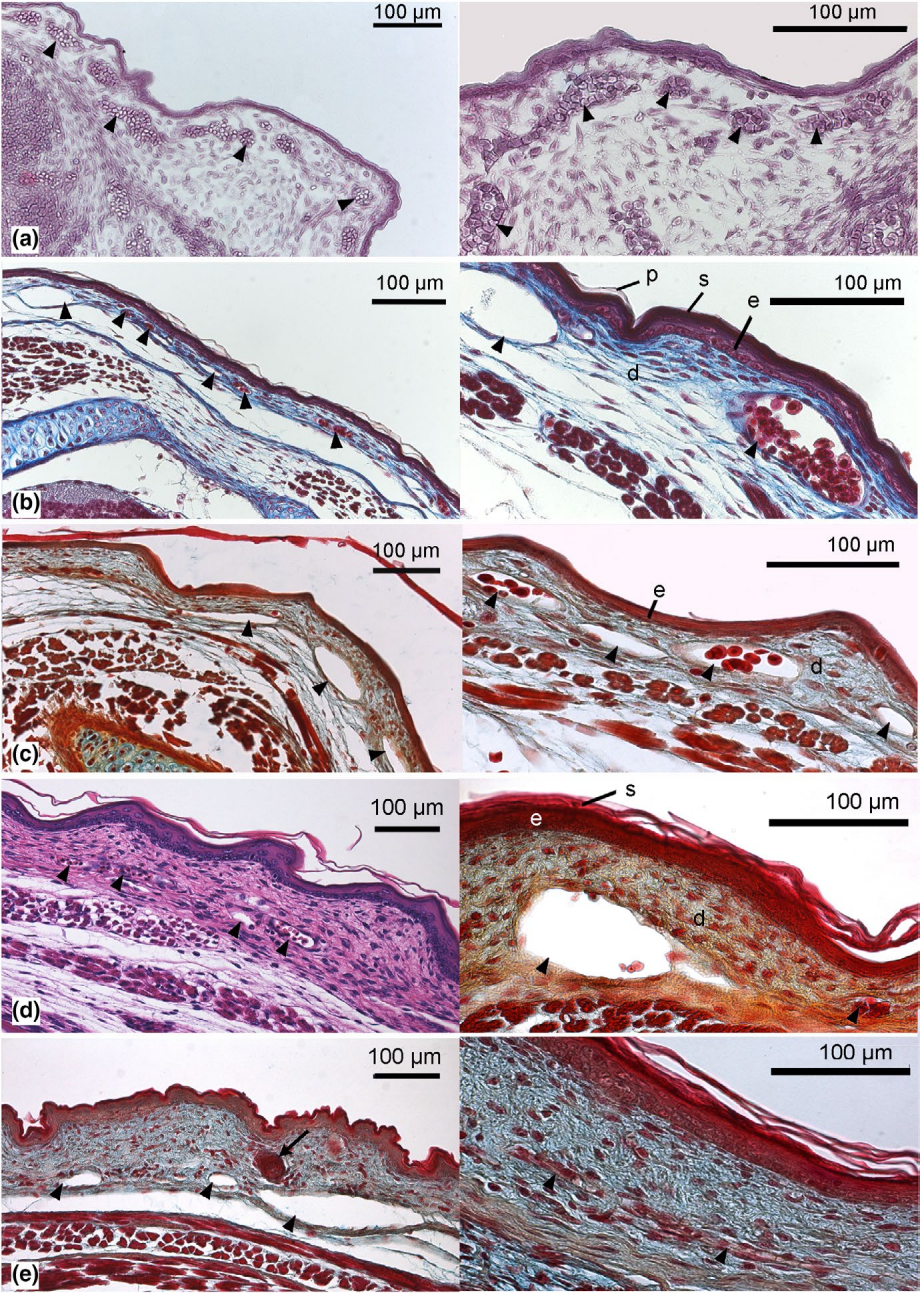
Histological sections comparing the structural skin development of *Monodelphis domestica* shortly before birth and in the postnatal period: 13 dpc (a), neonate (b), 2 dpn (c), 4 dpn (d), and 7 dpn (e). In the newborn and 2‐day‐old *M. domestica*, capillaries (arrowheads) are numerous and closely associated with the epidermis. A lower capillary density and increasing diffusion distance become noticeable with progressive age. Up to the age of 4 days, the outer layer of the epidermis is formed by the periderm. An additional thin layer of keratin (stratum corneum) is visible between the periderm and the basal region of the epidermis (see b, right side with higher magnification). First epidermal placodes appear by day 7 (indicated by the arrow). d, dermis; e, epidermis; p, periderm; s, stratum corneum. Magnification: Left photographs: ×200; right photographs: ×400.

**FIGURE 6:**
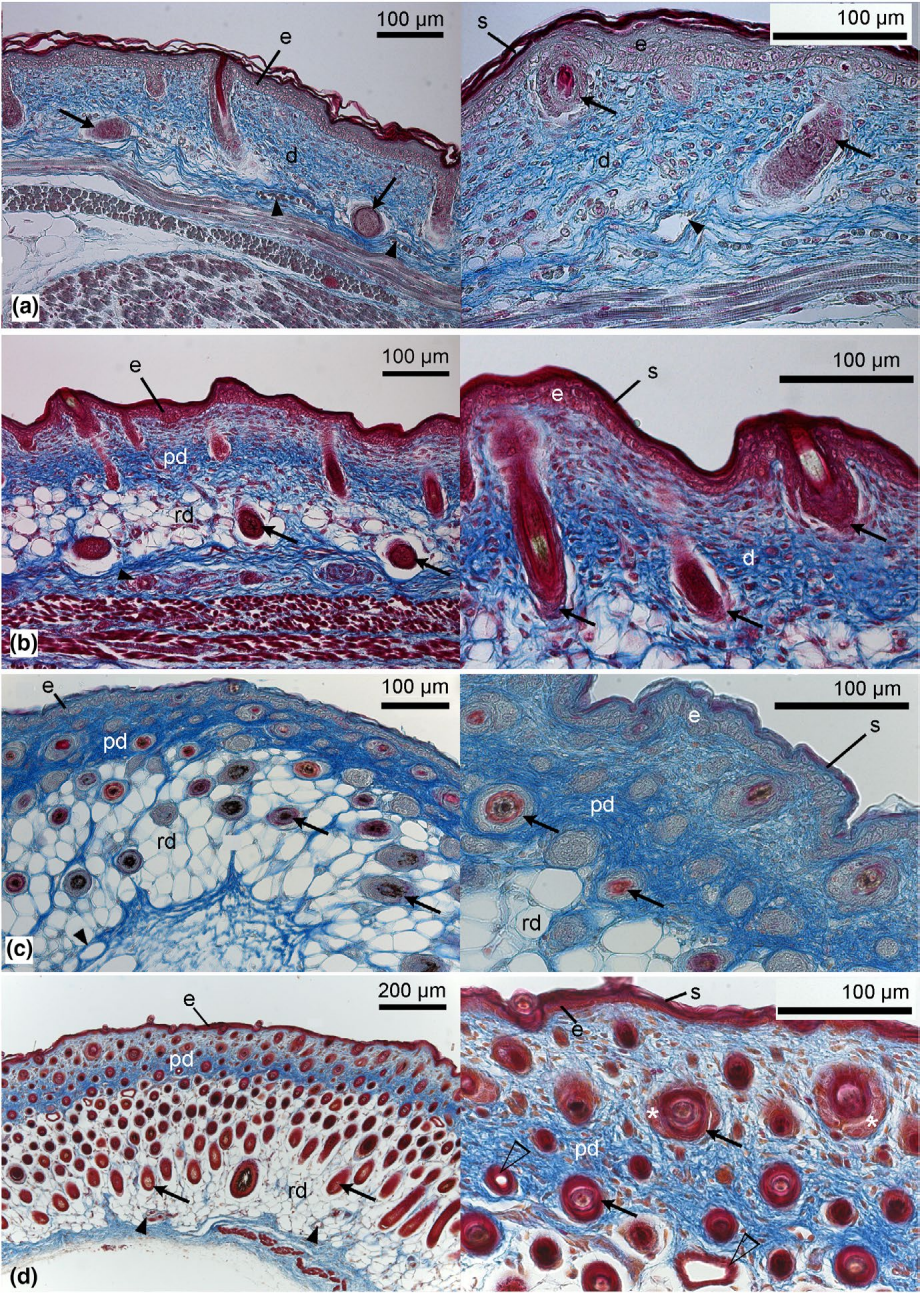
Histological sections comparing the structural skin development of *Monodelphis domestica* in the middle postnatal period: 11 dpn (a), 14 dpn (b), 21 dpn (c), and 28 dpn (d). Capillaries (filled arrowheads) become less numerous, and the diffusion distance between cutaneous capillaries and the skin increases further. Epidermal placodes and developing hair follicles (arrows) become more numerous by day 11. Starting by day 11, but more pronounced by day 14, the dermis differentiates into papillary and reticular layers. A multilayer of keratin (stratum corneum) forms the outer rim of the epidermis, providing cover and protection for the skin. The epidermis increases in thickness, reaching its maximal depth by day 21. The formation of hair follicles progresses, and mature hair follicles with cells of sebaceous glands (asterisk) and first sweat glands (open arrowhead) are visible by day 28. d, dermis; e, epidermis; pd, papillary dermis; rd, reticular dermis; s, stratum corneum. Magnification: Left photographs: ×200 (a–c), ×100 (d); right photographs: ×400.

**FIGURE 7:**
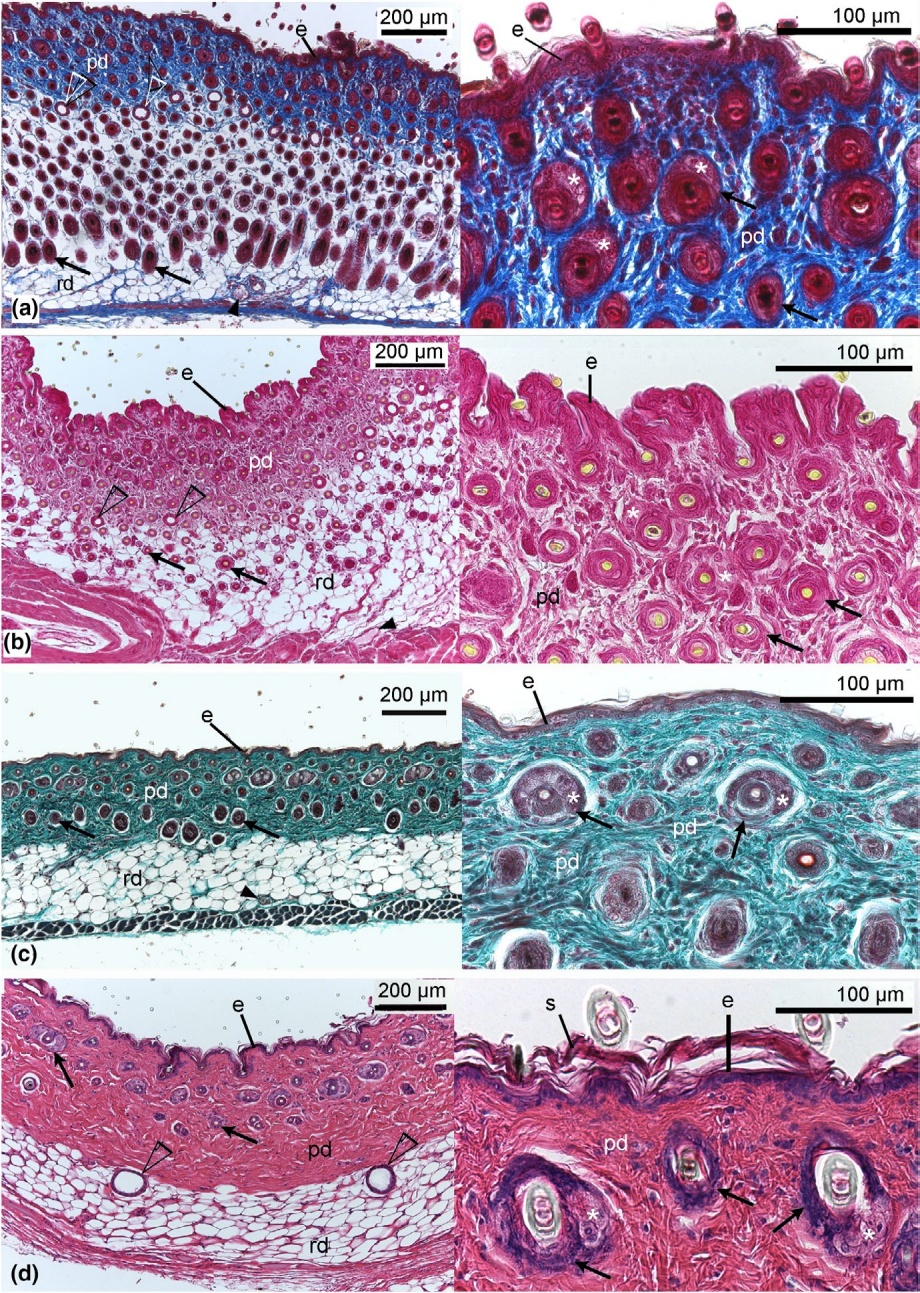
Histological sections comparing the structural skin development of *Monodelphis domestica* in the late postnatal period: 35 dpn (a), 49 dpn (b), 57 dpn (c), and adult (d). The highest hair follicle volume density during the entire skin development can be seen by day 35. At this time, the pelage is fully developed. Mature hair follicles (arrows) with sebaceous glands (asterisk) and numerous small sweat glands (open arrowhead) are present, indicating the onset of thermoregulation. Cutaneous capillaries (filled arrowheads) are rare and found only in the proximal part of the dermis. The epidermis becomes successively thinner, reaching a final depth of 14 μm in the adult skin. In the matured skin, the density of hair follicles decreases. Hair follicles are confined to the papillary dermis, and sweat glands are located on the border of the papillary and reticular dermis. e, epidermis; pd, papillary dermis; rd, reticular dermis. Magnification: Left photographs: ×100; right photographs: ×400.

**FIGURE 8:**
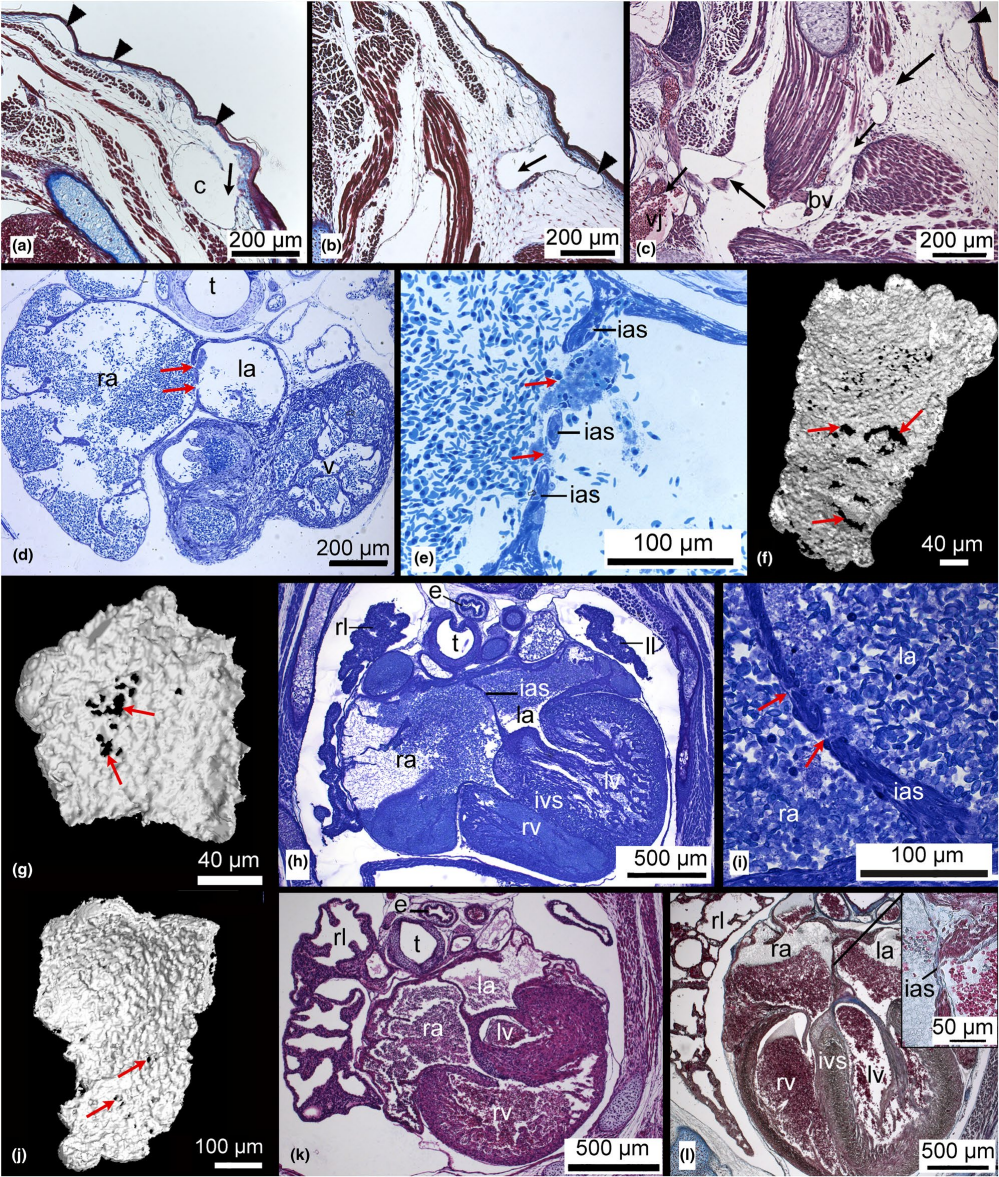
Light micrographs of the cardiorespiratory system and 3D reconstruction of the interatrial septum of the neonate (a–f), 2‐day‐old (g), 4‐day‐old (h–j), 7‐day‐old (k), and 11‐day‐old (l) *Monodelphis domestica*. In the newborn gray short‐tailed opossum numerous subepidermal capillaries (indicated by arrowheads) facilitate cutaneous gas exchange. They communicate with deeper‐lying capillaries running through the dermis (arrows), indicating a transport of blood to and from the cutaneous capillaries (a, b). These capillaries merge into larger blood vessels, which are drawing from peripheral regions towards the venous system (c). The heart of the newborn was immature with a fenestrated interatrial septum (d–f), permitting communication between the left and right sides of the heart (interatrial opening indicated by red arrows). By day 2, there are still small interatrial fenestrated openings in the thin septum (g). In the 4‐day‐old young, the interatrial septum is nearly complete (h–j). In the 7‐day‐old young, the heart is fully subdivided into left and right atria and ventricles (k). By 11 days the heart is muscular, and interatrial and interventricular septa increase in thickness (l). bv, blood vessel; c, capillary; e, esophagus; ias, interatrial septum; ivs, interventricular septum; la, left atrium; ll, left lung; lv, left ventricle; ra, right atrium; rl, right lung; rv, right ventricle; t, trachea.

**FIGURE 9:**
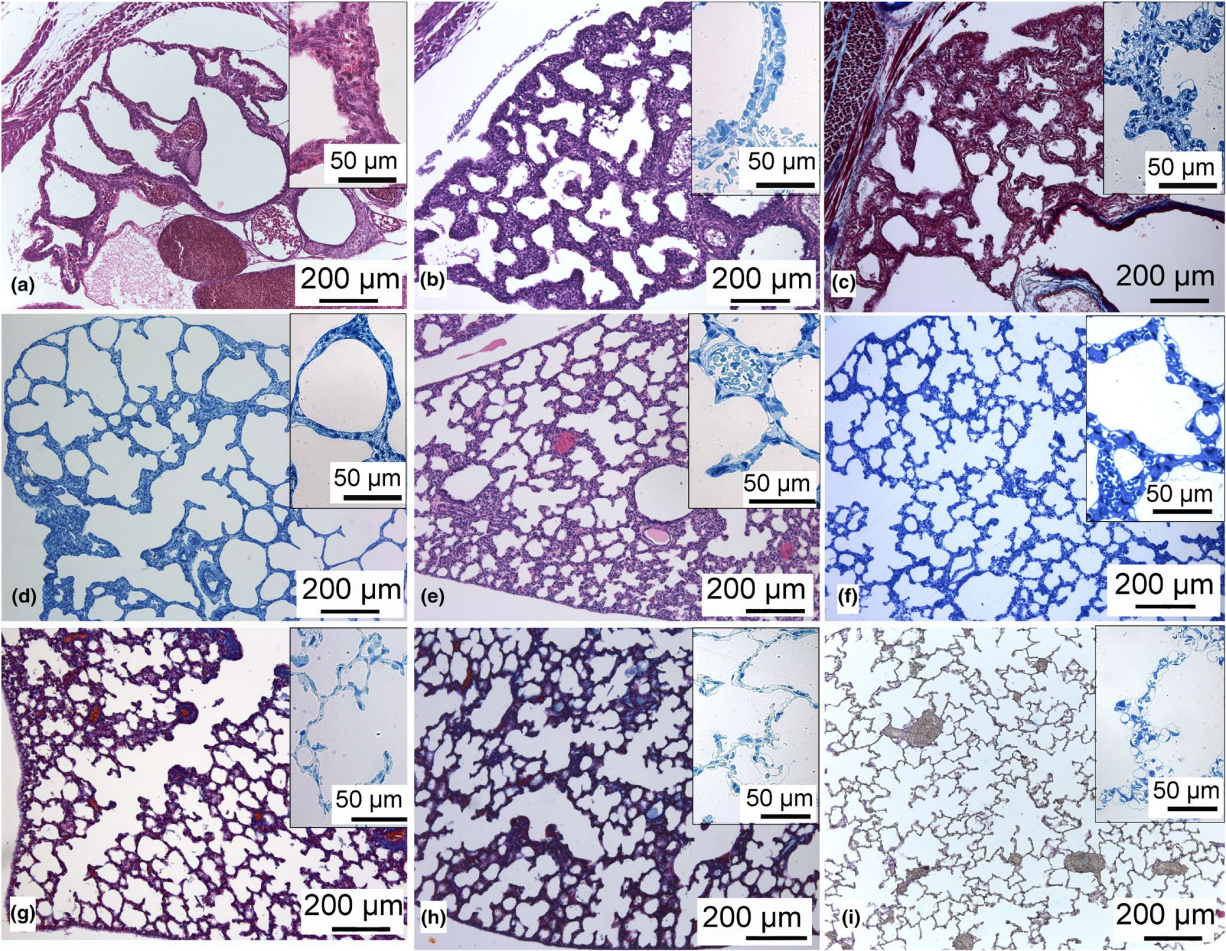
Postnatal lung development of *Monodelphis domestica*. Histological sections of a neonate (a) and by day 7 (b), 14 (c), 21 (d), 28 (e), 35 (f), 49 (g), 57 (h) and in the adult (i). The lung in the neonate *M. domestica* is at the canalicular stage. The bronchial tree is simple and consists of short wide lobar bronchioles supplying large terminal air spaces (a). During the first postnatal week, the air spaces become more and more subdivided by septal ridges. By day 7 (b) the lung enters the saccular period, characterized by a double capillary septum (c). Until day 28, the lung is still at the saccular stage, and newly formed septa are still double capillary (d–f). Alveolarization starts by day 28 (f), and by day 35 (g), the alveolar period, characterized by the presence of a single capillary bed, is fully attained. During the late postnatal development, alveolarization continues until the adult mature lung structure is established (g–i). Insets show the airspace septa in a higher magnification.

**FIGURE 10:**
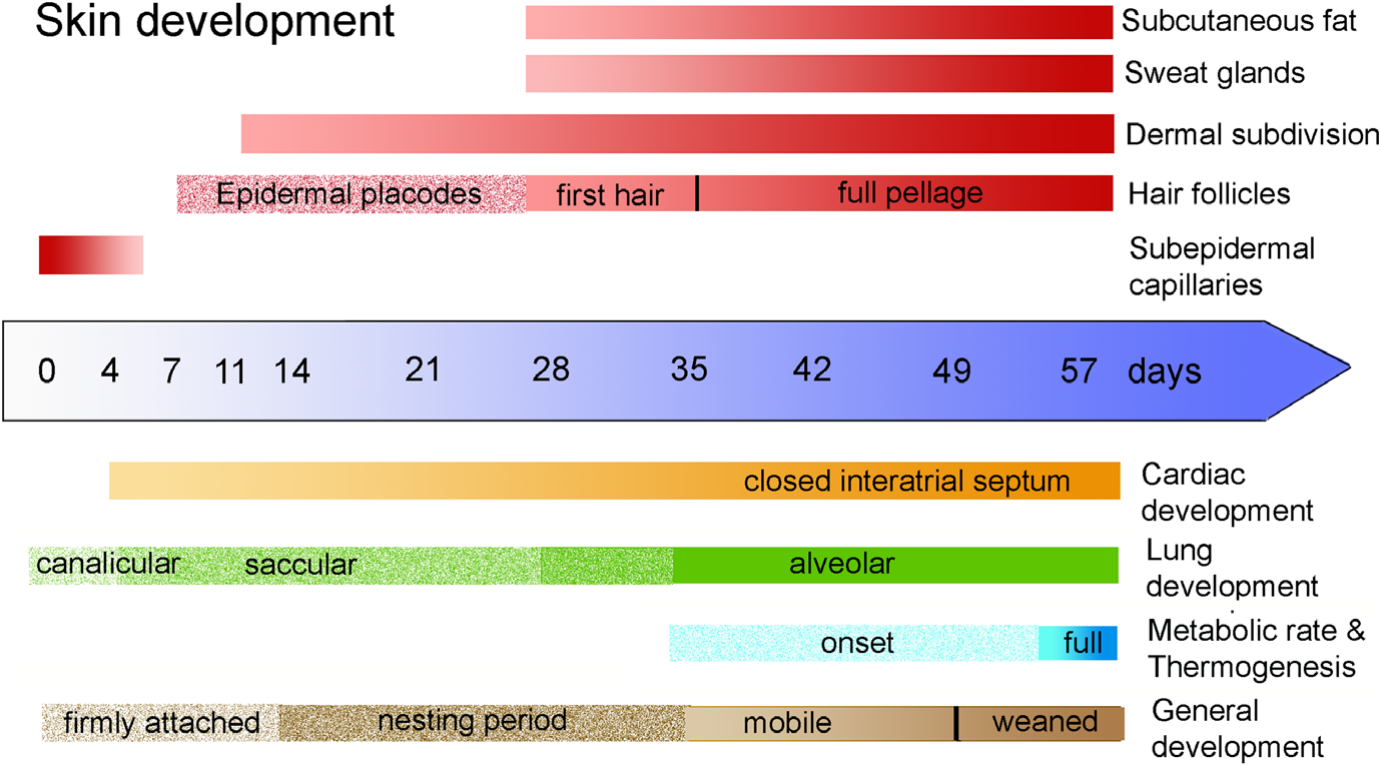
Timeline graph summarizing the important steps in cutaneous, pulmonary, cardiovascular, and general development in *Monodelphis domestica* during the postnatal period.

In the consecutive 4 days, the skin thickness nearly doubles to 107 ± 8 μm, due to an increase in dermal (79 ± 8 μm) and epidermal (23 ± 2 μm) layers. The periderm was discernible up to day 4; after that time, the stratum corneum formed the outermost layer of the epidermis. At day 2, cutaneous capillaries are still densely located in the dermis (Figures 3k and 5c). The capillary volume density of the skin is 0.27 ± 0.02, similar to that of newborns, but the diffusion distance increases to 35 ± 2 μm. By day 4, cutaneous capillaries can be seen less often; the capillary volume density of the skin decreases to 0.11 ± 0.02 (Figure 3l–n). The diffusion distance increases furthermore to 69 ± 5 μm (Figure 5d). This resulted mainly from newly formed connective tissue layers in the dermis between the capillaries and the thickening epidermal layer.

By day 7, the thickness of the entire skin measures 117 ± 6 μm (Figure 5e). The epidermis starts to show the various layers characteristic of the epidermis of the mature gray short‐tailed opossum. The stratum basale, with one layer of amorphous cells, the stratum spinosum, composed of approximately four layers of flattened cells, and the stratum corneum, formed by several layers of keratinized cells, are distinguishable. With the increasing thickness of the dermal and epidermal layers also the diffusion distance between the external surface and the most superficial capillaries also increases to 87 ± 1 μm. By this time, follicle primordia (epithelial placodes), the first signs of hair formation, can be seen (Figure 5e).

By day 11 (Figure 6a), the skin thickness increases to 178 ± 1 μm. This is due to the formation of additional connective tissue in the dermal layer. A subdivision of the dermis starts. In addition to the papillary layer, which consists of dense connective tissue, a reticular layer, consisting of loose connective tissue, is formed. The increase in thickness of the epidermis to 34 ± 5 μm is primarily due to an increase in the thickness of the spinous layer. As a result, the diffusion distance between capillaries and the skin surface measures 131 ± 12 μm, and cutaneous capillaries can be found mostly in the proximal part of the dermis with a low capillary volume density of 0.04 ± 0.00.

By day 14, the skin measures 243 ± 4 μm (Figure 6b). The dermis consists of papillary and reticular layers of similar thickness. The number of follicle primordia increases, indicating the progressing formation of hair follicles.

By 21 days, the skin thickness increases furthermore to 469 ± 9 μm (Figure 6c). The dermis consists of a thick reticular layer (320 ± 7 μm) with loose connective tissue and a thinner papillary layer (123 ± 1 μm) with dense connective tissue. The epidermis is getting thinner (21 ± 1 μm). A marked rise in the hair follicle volume density of the skin from 0.16 ± 0.01 by day 14 to 0.37 ± 0.01 by day 21 indicates the accelerated formation of hair follicles.

By day 28, the skin measures 610 ± 16 μm in thickness (Figure 6d). The major part of the skin is comprised of a thick reticular dermal layer (403 ± 7 μm). A thinner papillary dermal layer (189 ± 12 μm) is adjacent, and the thin epidermis (14 ± 1 μm) forms the boundary of the skin. Cutaneous capillaries are rare and are found only in the proximal part of the dermis. From this time, the value of capillary volume density stabilizes at a value of 0.01 ± 0.00 (see Table 2). The volume density of hair follicles in the skin is high (0.36 ± 0.01) by day 28, and the first hairs penetrate the skin. Fine dark fur appears at the back. By day 28, mature hair follicles with cells of sebaceous glands can be seen, and the first sweat glands appear. At this time, a subcutaneous layer of lipid cells (adipose tissue) could be observed.

**TABLE 2:** Skin development and thermoregulation in marsupial, monotreme, and placental mammals.

Species	Neonate				Full pelage (days)	Thermoregulation (days)		References
	Body‐weight (g)	Total skin thickness (μm)	Epidermal thickness (μm)^a^	Diffusion distance (μm)		Onset	Full	
Marsupialia								
*Dasyurus viverrinus*	0.012	36	9	9	70			Ferner (2021a)
*Dasyurides byrnei*					76	85–90	105–106	Geiser et al. (1986)
*Monodelphis domestica*	0.11	58	16	27	35			This study
*Didelphis virginiana*	0.13		43		60		70	Tyndale‐Biscoe and Renfree (1987)
*Isoodon obesulus*	0.18	75	19	41	49			Collins (1973)
*Perameles nasuta*	0.24	87	22	60				Ferner (2018)
*Phascolarctos cinereus*	0.5	115	22	45	168			Tyndale‐Biscoe and Renfree (1987), Ferner (2018)
*Trichosurus vulpecula*	0.2–0.32	89	24	37	117	130–133	140–143	Gemmell and Cepon (1993)
*Potourus tridactylus*	0.33	83	19	35	90		103	Gemmell et al. (1987)
*Bettongia gaimardi*	0.30				84	70	84	Rose and Kuswanti (2004)
*Setonix brachyurus*	0.35		30	45	130–145	100	120	Shield (1966) and Makanya et al. (2007)
*Macropus rufogriseus*	0.45				100–125		140	Loudon et al. (1985)
*Macropus eugenii*	0.37				180	140–180	180	Setchell (1974)
Monotremata								
*Ornithorhynchus anatinus*	0.3–0.4	75	26	47	<105			Ferner (2018)
Placentalia								
*Mesocricetus auratus*	1.8–2.1	243	28	‐	9–10	10	19	Hissa (1968) and Ferner (2018)
*Rattus rattus*	4.5–6	130	29	‐	14–16	1	15–16	Gerrish et al. (1998)
*Mus musculus*	1.5			‐	15	1	20	Szdzuy and Zeller (2009)
*Macroscelides proboscideus*	6–8	605	29	‐	0		0	Szdzuy (2006)

^a^ Epidermis, together with stratum corneum and periderm, if presented.

By 35 days, the skin thickness reaches a maximum of 726 ± 11 μm (Figure 7a). The volume density of hair follicles in the skin has 0.41 ± 0.01, the highest value of all developmental stages. The young are completely furred now. Numerous sweat glands can be seen at the border between the reticular and the papillary layers of the dermis.

The later postnatal skin development in the gray short‐tailed opossum from day 49 to adult (Figure 7b–d) is characterized by a decrease in total skin thickness resulting from the thinning of the reticular dermal layer (from 377 ± 17 to 274 ± 61 μm). The volume density of hair follicles in the skin is decreasing with age from 0.30 ± 0.01 to 0.16 ± 0.03. From day 57, hair follicles can be seen solely in the papillary layer of the dermis. The volume density of sweat glands in the skin increases in the 49‐ and 57‐day‐old animals but decreases in adults (see Table 1).

The measurements and morphometric results for the skin development of *M. domestica* are summarized in Table 1.

### 3.3 Structural development of the cardiac and respiratory system

In the gray short‐tailed opossum, numerous, partly elongated, cutaneous capillaries are located close to the epidermis (see Figures 2, 3, and 5), forming a large area for gas exchange. Deeper‐lying capillaries are running through the dermis, indicating a transport of blood to and from the cutaneous capillaries (Figure 5i). These capillaries merge into larger blood vessels that are drawing from peripheral regions towards the venous system (Figure 8a–c). The situation persisted in the 2‐day‐old young (Figure 5k) and to a lesser extent in the 4‐day‐old gray short‐tailed opossum (Figure 5m).

In the heart of the newborn gray short‐tailed opossum, a thin, fenestrated septum was found between the right and the left atrium (Figure 8d–f). A 3D reconstruction of the interatrial septum reveals that the fenestrations in the septum vary in size. The number of openings varies from one to five in single sections through the interatrial septum.

By day 2, there are still small interatrial fenestrated openings in the thin septum (Figure 8g). However, they are reduced in number and size. In the 4‐day‐old young, the interatrial septum fenestrated openings are barely recognizable, and the interatrial septum is nearly complete (Figure 8h–j). By 7 days, the heart is fully subdivided into left and right atria and ventricles (Figure 8k). At the age of 11 days, the heart becomes increasingly muscular, and interatrial and interventricular septa increase in thickness (Figure 8l). The heart matures structurally; cardiac valves develop and become more pronounced.

The pulmonary circulation of the gray short‐tailed opossum is established already at birth, and the lungs are working to facilitate gas exchange. At birth, the respiratory system of *M. domestica* is highly immature. The lungs of the neonate consist of large terminal air spaces and primitive airways characteristic of the late canalicular stage (Figure 9a). The terminal air spaces are lined with respiratory epithelium (type 1 and type 2 cells) interspersed with numerous respiratory capillaries (Figure 9a inset). The large terminal air spaces open from short lobar bronchi. During the first postnatal week, the lungs become more compartmentalized by septal ridges. By 7 days, the terminal air spaces are considerably smaller and more numerous (Figure 9b). The air space septa consist of a double capillary bed now, indicating the saccular period of lung development (Figure 9b inset). The process of septation of the lung parenchyma continues for the next 3 weeks. The newly formed double capillary septa become progressively thinner (Figure 9c–e insets). By 28 days, the first alveoli appear, and the microvascular maturation transforms double to single capillary septa, indicating the start of the alveolar period of lung development (Figure 9e inset). By 35 days, the alveolar period is fully attained, and single capillary septa are prevailing (Figure 9f). The continued alveolarization leads to the final adult lung structure (Figure 9g–i) consisting of typical acini characteristic of the adult mammalian lung.

## 4 DISCUSSION

Newborn marsupials are distinguished from eutherian neonates by their relative immaturity and small size at birth. They spend most of their development postnatally attached to one of the maternal teats, within or without a pouch (Ferner et al., 2017; Renfree, 2006; Simpson et al., 2011; Tyndale‐Biscoe & Renfree, 1987).

The newborn gray short‐tailed opossum resembles neonates of other didelphids, such as the Virginia opossum (*Didelphis virginiana*), and peramelids (Cutts et al., 1978; Hughes & Hall, 1988). Hughes and Hall (1988), in their review of the structural adaptations of newborn marsupials, considered didelphids (G2) to be more developmentally advanced than dasyurids (G1) and less developed than macropodids (G3) at birth. Many factors influence postnatal growth and development, but a general trend is for smaller species to develop more rapidly than larger species (Zullinger et al., 1984). This relationship holds for marsupials in which the young of small species, such as *M. domestica* (body weight 80–120 g) are independent by day 57 postpartum, whereas those of large marsupials, e.g., Bennett's wallaby (*Macropus rufogriseus*; body weight 14–19 kg), are not independent until day 360 (Szdzuy & Zeller, 2009).

### 4.1 Skin development

In the newborn gray short‐tailed opossum, the thickness of the skin measures 58 μm. In the considerably smaller, less mature newborn eastern quoll (*Dasyurus viverrinus*; body weight 12.5 mg) the skin measures only 36 μm but reaches a thickness of 54 μm, comparable to that of the newborn gray short‐tailed opossum, by day 3 (Ferner, 2021a). In contrast, more mature marsupial neonates, such as the Southern brown (*Isoodon obesulus*), long‐nosed bandicoot (*Perameles nasuta*), brush‐tail possum (*Trichosurus vulpecula*), long‐nosed potoroo (*Potorous tridactylus*), koala (*Phascolarctos cinereus*), brush‐tailed rock wallaby (*Petrogale penicillata*), black‐striped wallaby (*Notamacropus dorsalis*), and red‐necked pademelon (*Thylogale thetis*), have a greater skin thickness (83–186 μm) (Ferner, 2018). Similar to marsupials, the newly hatched monotreme platypus (*Ornithorhynchus anatinus*) has a skin thickness of 75 μm (Ferner, 2018). In contrast to marsupials and monotremes, eutherian neonates always have a thicker skin, and hair follicles are developing in altricial species (e.g., golden hamster [*Mesocricetus auratus*], skin thickness: 243 μm) or are already present in precocial species (e.g., short‐eared elephant shrew [*Macroscelides proboscideus*], skin thickness: 605 μm) (Ferner et al., 2017).

The skin development in the gray short‐tailed opossum appears to be similar to that described in other marsupial species studied so far (Armstrong & Ferguson, 1995; Ferner, 2018, 2021a; Krause et al., 1978; Lyne, 1970; Lyne et al., 1970; Makanya et al., 2007; Pralomkarn et al., 1990). In the early postnatal period, up to 4 days, a 1–2 μm thick keratinized membrane, the periderm, is present in the gray short‐tailed opossum. Such a periderm (also called ‘epitrichium’) has also been described in the Virginia opossum (*D. virginiana*) (Krause et al., 1978), the brush‐tailed possum (*T. vulpecula*) (Lyne et al., 1970), and the northern (*Dasyurus hallucatus*) (Pralomkarn et al., 1990) and eastern quoll (*D. viverrinus*) (Ferner, 2021a). The periderm is defined as “the superficial transient layer of epithelial cells of the embryonic epidermis.” In marsupials, the periderm is believed to provide a protective barrier that prevents desiccation during the first few days after birth and to offer immune protection (Hill & Hill, 1955; Krause et al., 1978; Pralomkarn et al., 1990; Smith & Keyte, 2020). Pralomkarn et al. (1990) state that the reduction and finally disappearance of the periderm might be due to a result of the exposure to ambient air. When the newborn marsupial moves from the aqueous environment of the uterus to the aerobic environment of the pouch, the intracellular fluid is lost from the periderm, and thus the outer layer of cells assumes the normal features of a cornified layer.

In eutherians, the periderm is observed prior to the formation of hair follicles and disappears when the hair shafts emerge through the epidermis (Hanson, 1947; Holbrook & Smith, 1981; Lyne & Hollis, 1972). Thus, in eutherians, the periderm is only present in utero and disappears with the eruption of hair. However, the correlation between hair shafts and the disappearance of the periderm, observed in eutherians, is not valid for marsupials, since the periderm disappears soon after birth, well before the appearance of hair follicles.

The epidermis, together with its outer layers, the stratum corneum and the periderm, forms a protective barrier. The thickness of the epidermal layers is 16 μm at birth. During the early postnatal period, the epidermis increases in thickness, reaching a maximum (34 μm) at day 11. This increase is the result mainly of an expansion of the stratum spinosum. After day 11, the epidermis undergoes a progressive thinning, resulting in an epidermal thickness of 14 μm in the adult gray short‐tailed opossum. A similar sequence of development has been reported for the epidermis of the Virginia opossum (*Didelphis viginiana*) (Krause et al., 1978) and the brush‐tailed possum (*T. vulpecula*) (Lyne et al., 1970). In these species, the epidermis shows a marked increase in thickness during the first 3 weeks of postnatal life and subsequently undergoes a progressive thinning. As in *M. domestica*, the epidermis of the adult *D. virginiana* and *T. vulpecula* measures only about 14 and 15 μm in depth. The period of rapid development of the epidermis, in all three species, coincides with the development of the first population of hair follicles. First hair follicles can be seen in the gray short‐tailed opossum at day 7. Initial development of hair follicles by day 7 has been reported also for the Virginia opossum (*D. viginiana*) (Krause et al., 1978). In the northern quoll (*D. hallucatus*), epidermal placodes appeared by day 23, and hair follicles were present by day 30 (Pralomkarn et al., 1990). Similarly, all species show continued differentiation and development of hair follicles during the period of decreasing thickness of the epidermis. An increase in epidermal thickness prior to and/or during the period of follicular differentiation also occurs in eutherian species (Lyne & Heideman, 1959).

The total skin thickness in the gray short‐tailed opossum increases from 58 μm at birth to 726 μm by day 35, when the pelage is fully developed. The thickening of the skin accounts mainly for the development of the dermis by acquiring more collagen. The subepithelial dermal region becomes more cellular than the deep dermis. Starting from postnatal day 11, the dermis becomes a two‐layer structure, the more superficial portion of papillary dermis and the deeper portion of reticular dermis. The same process of structural transformation of the dermis has been described by Armstrong and Ferguson (1995). The gray short‐tailed opossum has a subcutaneous fat layer from day 28 on. In this respect, the skin of *M. domestica* resembles human skin but differs from that in rats and mice, which do not have this adipose layer or develop it later during adult life (Armstrong & Ferguson, 1995).

In neonates of altricial eutherians (e.g., the golden hamster, *Mesocicetus auratus*), the skin is thick (243 μm), and a few hair follicles, sweat, and sebaceous glands are already present in the dermal tissue. The skin structure resembles the skin of the 14‐day‐old gray short‐tailed opossum (Ferner et al., 2017). In precocial eutherian neonates (e.g., the short‐eared elephant shrew, *M. proboscideus*), the skin is considerably thicker (605 μm), and a great number of hair follicles associated with sebaceous glands and sweat glands are present in a massive dermal layer. Hair follicles of epidermal origin deeply penetrate into the dermis. Hair is protruding through the epidermal surface and covers the animal in dense fur (Ferner et al., 2017). The skin structure and thickness of the newborn short‐eared elephant shrew resemble the skin of the 35‐day‐old gray short‐tailed opossum. Information about skin development and thermogenesis in mammals is provided in Table 2.

The developmental milestones of skin development in *M. domestica* are summarized and compared to pulmonary, cardiovascular, and general development during the postnatal period in Figure 10.

### 4.2 Cutaneous gas exchange

In some dasyurid marsupial newborns, gas exchange through the skin accounts for almost 100% of total gas exchange. In these smallest of all marsupial neonates, transcutaneous gas exchange is obviously a requirement due to the inefficient pulmonary ventilation (no breathing movements and volume changes) attributable to the poor neuro‐muscular development (Frappell & Mortola, 2000). Even in the relatively large newborn tammar wallaby (*M. eugenii*; ~350 mg), gas exchange across the skin accounts for 33% of total gas exchange (MacFarlane et al., 2002; MacFarlane & Frappell, 2001). In the newborn gray short‐tailed opossum, which represents an intermediate developmental stage, the skin might possibly account for 50%–60% of total gas exchange (Ferner, 2018).

In all neonate marsupials investigated so far, the epidermis of the skin was poorly developed with a thin stratum corneum and no hair follicles and provides only a weak barrier to transcutaneous gas exchange (Ferner, 2018; Krause et al., 1978; Lyne et al., 1970; Makanya et al., 2007; Pralomkarn et al., 1990). The presence of numerous capillaries in the dermal layer of all marsupial neonates and the monotreme hatchling supports the notion that cutaneous exchange in newborn marsupials and probably also newly hatched monotremes might be commonplace (Ferner, 2018).

Skin respiration is often significant and crucial in invertebrates and lower vertebrates (Feder & Burggren, 1985; Mortola, 2015). However, for birds and mammals, the skin is not an important route for gas exchange. Cutaneous gas exchange in higher vertebrates is limited by the poor gas diffusion properties of the skin tissues, the low surface area to mass ratio resulting from large body size, and the high metabolic demands (Makanya et al., 2007; Mortola, 2015). Cutaneous respiration in marsupial offspring is made possible by a high surface‐to‐volume ratio (due to small body size) and by a low specific metabolic rate (favoring the diffusion depth of oxygen) (Singer, 2004).

Young mammals and birds are often born or hatched with thin skin, richly supplied with blood and lacking fur or feathers, at least in altricial neonates or hatchlings (Ferner et al., 2009, 2010, 2017; Szdzuy & Zeller, 2009). Therefore, cutaneous gas exchange is more important in the developing young than it is in the adults.

The near‐term embryo of the gray short‐tailed opossum has the highest capillary volume density in the skin (Vvc = 0.44), and the 3D reconstructions of cutaneous capillaries reveal a dense net of cutaneous capillaries all over the body of the embryo (see Figure 3). The subepidermal capillary network resembles the vascularization seen in adult anurans (Saint‐Aubain, 1982). Although these findings have no physiological consequences for the later postnatal gas exchange via the skin, the high capillarity of fetal skin could be a pre‐adaptation to the gas exchange in the postnatal period or might play a role in maintaining the water balance of the amniotic fluid. In humans, the amniotic fluid is created by the flow of fluid from the fetal lung and bladder but also arises as a transudate of plasma, either from the fetus through non‐keratinized fetal skin or from the mother across the uterine decidua and/or placenta surface (Beall et al., 2007).

The thin skin of the newborn gray short‐tailed opossum, with short diffusion distances (blood‐air‐barrier) as well as a high capillary volume density, provides excellent preconditions for effective cutaneous gas exchange. The skin diffusion barrier of the newborn *M. domestica* measures 27 μm, which is considerably thicker than the blood‐air barrier in the terminal air spaces of the neonate (605 nm) or in the alveoli of adults (227 nm) (Szdzuy et al., 2008). However, the thickness of the skin diffusion barrier is similar to that reported from other marsupial neonates (23–65 μm) and other species with cutaneous gas exchange as well (amphibians: 31–42 μm; folded wing of the fruit bat *Epomorphorus wahlbergi*: 27 μm) (Ferner, 2018; Makanya et al., 2007). A significantly lower diffusion distance of 9 μm was reported from the newborn eastern quoll (*D. viverrinus*) (Ferner, 2021a) and the epidermal thickness (9.8 μm) in the stretched wing web membrane of the fruit bat (*E. wahlbergi*) (Makanya & Mortola, 2007). However, even diffusion distances ranging from 23 to 65 μm should be sufficient for gas exchange, since cutaneous oxygen diffusion for the supply of underlying tissue can reach depths of 250–400 μm (Stücker et al., 2002).

With 0.25, the capillary volume density in the skin of the newborn gray short‐tailed opossum was lower than in the near‐term embryo but still highest in the postnatal period.

It is lower than that present in the newborn eastern quoll, *D. viverrinus* (0.33), but higher compared to more mature marsupial neonates (0.09–0.13) (Ferner, 2018, 2021a). In the gray short‐tailed opossum, the capillary density decreased rapidly during the first four postnatal days to 0.11. At the same time, the diffusion distance between cutaneous capillaries and the air increased to 69 μm due to newly formed tissue in the outward‐growing epidermal and dermal layers. The interatrial septum is nearly complete by this time, and cutaneous gas exchange ceases. From this time on, cutaneous capillaries decrease and serve the supply of the skin and play a role in hemodynamic and thermoregulatory regulation, but play no role in cutaneous respiration anymore.

The decrease in capillary density, starting by day 4, might be explained by vessel regression (for review see Korn & Augustin, 2015). Apoptosis of endothelial cells and several molecular mediators might be involved in the regression process of the superficial capillaries. Vessel remodeling leading to pruning and regression is a common phenomenon during embryonic and postnatal development of the rat (Hughes & Chang‐Ling, 2000) and mouse (Franco et al., 2015; Udan et al., 2013). Furthermore, a reduction of blood flow toward the skin following cardiac remodeling might play a role (Ando & Yamamoto, 2009).

Animals with percutaneous gas exchange often live in a moist environment because they face a high risk of excessive water loss via the skin (Mortola, 2015). The humidity of the pouch helps the marsupial neonate to circumvent this problem. However, there are ‘pouch‐less’ marsupial species among the dasyurids (e.g., *D. viverrinus*) and didelphids (*M. domestica*) that lack the true, permanent pouches seen in other species and where the young are exposed to the air (Tyndale‐Biscoe & Renfree, 1987; Woolley et al., 2002). In *M. domestica*, the lack of a pouch seems to be compensated by maternal behaviour since the mother is often observed crouched over the litter providing shelter and heat and stays most of the time in a nest (Fadem et al., 1982). But when the mother has to move (e.g., foraging) the young might have a lower body temperature compared to marsupial pouch young, which would be associated with a lower metabolic rate and better diffusion conditions. There seems to be a correlation between being ‘pouch‐less’ and high rates of cutaneous gas exchange in marsupials. The advantage of being exposed to an infinite pool of oxygen obviously outweighs the greater risk of desiccation.

### 4.3 Circulatory and cardiac system

During the early postnatal period, the skin of the gray short‐tailed opossum possesses a well‐developed capillary network for cutaneous gas exchange. Besides the superficial subepidermal capillaries, the blood transport to and from the skin, as well as communication with the systemic circulation, is important and has been described for the eastern quoll (*D. viverrinus*) (Ferner, 2021a). The capillaries close to the epidermis communicate with deeper‐lying capillaries in the dermis, indicating a transport of blood to and from the subepidermal capillaries. These capillaries join into larger blood vessels that communicate with the arterial and venous systems.

Since cutaneous respiration is only a temporary solution in the early postnatal period in marsupials, a temporary circulatory arrangement for gas exchange via the skin is meaningful. Adult marsupials have a pulmonary and systemic circulation similar to eutherians, and in spite of birth at an earlier stage of development, marsupials have similar cardiovascular changes to eutherians in the transition from intrauterine to extrauterine life (Runciman et al., 1995). In all mammals, the vascular communication, or “shunts” between the pulmonary and systemic sides of the heart, the foramen ovale and ductus arteriosus, permit both atria and both ventricles to operate in parallel in the fetus prior to the maturation of the pulmonary circulation after birth (Taylor & Weibel, 1981). The basic differences between eutherians and marsupials are that the cardiorespiratory system of marsupials has to operate for aerial respiration in a neonate of smaller body size (less than 1 g) and that the major period of development in marsupials occurs under higher O_2_ levels and lower CO_2_ levels (pouch) than in utero in the eutherian fetus (Baudinette et al., 1988).

In marsupials, the cardiovascular system must develop to a functional stage within a gestation period as short as 13.5 days in the case of *M. domestica*. The heart of the newborn gray short‐tailed opossum allows for communication between the left and right sides of the heart, provided by a fenestrated interatrial septum. An interatrial communication is a common phenomenon during the immediate postnatal period in marsupials and was described in the newborn tammar wallaby (*Macrous eugenii*), eastern quoll (*D. viverrinus*), brush‐tail possum (*T. vulpecula*), northern brown bandicoot (*Isoodon macrourus*), brush‐tailed rock wallaby (*P. penicillata*), and eastern gray kangaroo (*Macropus giganteus*) (Baudinette et al., 1988; Ferner, 2021a; Runciman et al., 1995). In developing eutherians, two central cardiovascular shunts, the ductus arteriosus and the foramen ovale, allow up to 90% of the blood from the right ventricle to pass into the systemic circulation (Rudolph, 1979). In the neonate *M. domestica*, investigated in this study, the ductus arteriosus was already closed. A patent ductus arteriosus, forming a communication between the pulmonary trunk and the proximal descending aorta, was described in several prenatal marsupial species (Runciman et al., 1995). The ductus arteriosus was constricted at 4 h after birth in the tammar wallaby (*M. eugenii*) and the lumen was occluded within 6 h of birth. Occlusion had occurred by 12 h after birth in the native cat (*D. viverrinus*), within 24 h in the brushtail possum (*T. vulpecula*), the northern brown bandicoot (*I. macrourus*) and long‐nosed bandicoot (*P. nasuta*), and the eastern gray kangaroo (*M. giganteus*) and within 16 h of birth in the eutherian mouse (*Mus musculus*).

In the most immature marsupial newborn, the eastern quoll, in addition to the interatrial shunt, a large interventricular communication persists for up to 3 days after birth (Ferner, 2021a; Runciman et al., 1995). From a hemodynamic point of view, the following line of reasoning could be relevant for cutaneous gas exchange in newborn marsupials: The venous return from the skin can easily pass through the interatrial connection (similar to the blood from the umbilical vein in the fetal circulation via the foramen ovale). Pulmonary vascular resistance is likely to be increased as long as the lung is not fully aerated (especially in daysurid neonates where the lung is not functioning for 3 days). With time, skin vessels become rarer, leading to an increase in peripheral resistance. This results in an increased blood pressure in the systemic circulation, which in turn reduces the right‐to‐left shunt via the interatrial connection. Thus, the hemodynamics in neonate marsupials resemble fetal eutherian circulation, and the skin may be considered a temporary “replacement placenta” until the lung is fully functional.

After the closing of the shunts in the early postnatal period, the pulmonary and systemic circulations are completely separated. All right cardiac output is directed to the lungs, and right and left ventricular volumes are balanced. From this time on, around 2–4 days after birth, cutaneous respiration ceases, and pulmonary respiration has to take over completely. The rapid maturation of the lung, in parallel, allows for the pulmonary system to be functional in order to meet the metabolic needs of the organism.

### 4.4 Lung development

There is a graduation of lung development ranging from canalicular to saccular stage among newborn marsupials (Hughes & Hall, 1988; Mess & Ferner, 2010). Generally, marsupial species with very low birth weight have lungs at birth consisting of a few tubular air spaces (G1; e.g., *D. viverrinus*), neonates with intermediate birth weights have lungs consisting of large terminal air spaces separated by thick primary septa (G2; e.g., *M. domestica*, *T. vulpecula*, *P. nasuta*); and the ones with the highest birth weights have further subdivided saccular lungs (G3; e.g., *Phascolarctos cincereus*, *P. penicillata*, *M. eugenii*) (Ferner, 2018, 2021b; Gemmell, 1986; Gemmell & Nelson, 1988; Gemmell & Selwood, 1994; Krause & Leeson, 1975; Runciman et al., 1996; Szdzuy et al., 2008).

The lungs of the neonate gray short‐tailed opossum are at the canalicular stage and consist of poorly subdivided large terminal air spaces which branch from a rudimentary bronchial tree (Ferner, 2024a; Ferner & Mahlow, 2023). Although the lung is structurally immature, the presence of surfactant proteins (SP‐A, SP‐B, and SP‐D) at the site of gas exchange at birth suggests that the surfactant system is fully functional (Orgeig et al., 2004) and the lung is working as the main gas‐exchanging organ.

The lung of the gray short‐tailed opossum develops rapidly. Between day 4 and 7, the lung enters the saccular stage of lung development, and the lung parenchyma becomes more and more subdivided, increasing the surface area for gas exchange. The pulmonary circulation develops before the bronchial tree is established (Ferner, 2024b). At the time the cutaneous respiration ceases, the lung is sufficiently developed to comply with the respiratory requirements of the growing organism. Between day 28 and 35, when the lung attains the alveolar stage, the gas exchange surface area increases significantly, and the metabolic rate rises noticeably (Ferner, 2024a; Szdzuy et al., 2008).

### 4.5 Thermoregulation

All marsupial young, including those of *M. domestica*, are born without hair and are incapable of maintaining a steady body temperature (Szdzuy & Zeller, 2009; Wacker et al., 2017). Because of their relatively large surface area, small marsupial young lose heat very rapidly when exposed to low ambient temperatures. However, the postnatal development of *M. domestica* (Figure 1) reveals a rapid increase in body size. The rates of heat and water loss decrease with growth because the surface‐to‐volume ratio decreases with increasing body size (Hulbert, 1988). The thyroid gland, which influences skin structure and the development of thermoregulation, is not present in newborn marsupials (Pralomkarn et al., 1990; Setchell, 1974). However, a functional thyroid gland is necessary for these developmental processes. Thyroid function, furring, and thermogenic capacity in the marsupial do not occur until approximately two‐thirds of pouch occupancy (Geiser et al., 1986; Setchell, 1974).

Initially, the thermogenic response is small, but gradually the maximal thermogenesis possible increases until it is similar to that of the adult. The mechanisms of endothermic thermogenesis are not fully understood, but there may be both shivering and non‐shivering thermogenesis (Hulbert, 1988; Jastroch et al., 2007). The development of thermoregulation was studied in two dasyurid marsupials, the fat‐tailed dunnart (*S. crassicaudata*) and the kowari (*Dasyuroides byrnie*). In the fat‐tailed dunnart (*S. crassicaudata*), thermoregulation developed in three stages. From day 0 to day 40, animals were unable to maintain a constant high body temperature during short‐term cold exposure. By day 60, animals maintained a high body temperature for some time but then entered torpor and could only rewarm passively when basking under a heat lamp. From day 90, they expressed prolonged torpor bouts and were able to rewarm endogenously (Wacker et al., 2017). The appearance of hibernation‐like adaptations in the postnatal period in marsupials as well as in the perinatal period in eutherians supports the hypothesis of a similarity between mammalian torpor and perinatal adaptations (Singer, 2004). Also, the kowari (*D. byrnie*) is not capable of thermogenesis until day 70–80 (Geiser et al., 1986). During the period of gradual attainment of thermogenic capacity, hair growth begins, and by the time full thermogenic capacity is reached, a complete pelage is present. In pouched marsupial species, the young do not leave the pouch until they are capable of endothermic regulation of their own body temperature (Hulbert, 1988).

In the gray short‐tailed opossum, the formation of hair follicles increases exactly when the young detach from the maternal teat (14–21 days) and the young are left in a nest. At this time, the skin resembles that of an altricial eutherian neonate.

The volume density of hair follicles in the skin reaches its maximum between 28 and 35 days, and first sweat glands and subcutaneous adipose tissue can be observed. This coincides with the time when the lung attains the alveolar period, the metabolic rate rises, the eyes and ears open, and the young start leaving the nest.

White adipose tissue (WAT) plays an essential thermoregulatory role by providing insulation and serving as an energy reservoir and endocrine signaling for the developing marsupial offspring. Marsupial WAT exhibits unique characteristics (diverse cell types, high metabolic activity, immunological crosstalk) that may influence its role in energy homeostasis and metabolic regulation (Lezama‐García et al., 2022). The immaturity of the thermoregulatory system of newborn marsupials bears similarities to preterm human neonates. Like newborn marsupials, preterm neonates are at a high risk of hypothermia due to their smaller body size and the “true” immaturity of effector systems (lack of isolating white and heat‐producing brown adipose tissue) (Singer, 2013). Similarly to marsupial neonates, preterm neonates have an increased permeability of the skin (resulting in evaporative heat loss) and a delayed increase in basal metabolic rate (Singer, 2013).

In many marsupial species, the time a full pelage is present coincides with the onset of thermoregulation (see Table 2; Szdzuy & Zeller, 2009). That supports the view that thermogenic capacity develops late in postnatal development and is correlated to furring and insulation.

This is also valid for eutherian mammals; however, it occurs earlier in postnatal development. Achievement of full thermogenic capacity correlates with the development of insulating fur in altricial species, and thermoregulation is already fully developed in completely furred precocial neonates (Table 2; Szdzuy et al., 2008).

The small size of altricial placental neonates threatens them with hypothermia and desiccation like marsupial neonates. Because the internal organs are further developed, they support the maintenance of vital functions and a short metabolic increase if required. However, this short‐term metabolic increase is always at the expense of growth (Hill, 1992). Most altricial eutherian neonates completely depend on their mother for warming and nourishment. They have poor thermoregulation, poor insulation, and limited heat production ability (Harjunpää & Rouvinen‐Watt, 2004). The golden hamster (*M. auratus*), for example, shows no endogenous thermoregulatory response to cold temperatures until days 7–10 the time when fur develops (Hissa, 1968). The main thermoregulatory mechanisms in very small altricial newborns are non‐shivering thermogenesis (via brown adipose tissue) and behavioral responses (huddling with nest‐mates, seeking a warmer place, or calling for their mother; Asakura, 2004; Bryant & Hails, 1975; Sundin & Cannon, 1980; Withers & Jarvis, 1980). Because of the dense fur and rapid growth as well as their thermoregulatory abilities, precocial neonates respond to cold by increasing heat production from very early on in life (Hackländer et al., 2002; Waldschmidt & Müller, 1988). A useful strategy is lowering thermal conductance by means of effective insulation and peripheral vasoconstriction to reduce heat loss in the cold (Hackländer et al., 2002). In larger precocial newborns, both non‐shivering and shivering thermogenesis can be found (Brück & Wünneberg, 1966).

### 4.6 Immune protection and wound healing

Skin development in marsupials is a fascinating area of study, particularly due to their unique reproductive strategies and the distinct characteristics of their skin compared to placental mammals. The skin of newborn marsupials is immature but adapted for life in the external environment. The thick outer layer of epidermal cells, the periderm or epitrichium, seems to offer immune protection, as a non‐sterile cut to the periderm leads to infection and death of the newborn (Smith & Keyte, 2020). The immune system of marsupial neonates is immature at birth, making them vulnerable to infections. However, they possess innate immune mechanisms that help protect them during this critical period.

The newborn's immune protection is bolstered by unusual adaptations involving both the neonate and the mother. Mechanisms arising from the mother include a very large variety of substances transferred to the young via maternal milk, including immunoglobulins, transferrin, lysozymes, neutrophils, and macrophages (e.g., Lefèvre et al., 2010; Renfree, 2010; Smith & Keyte, 2020). Pouch secretions have also been shown to include substances with demonstrated antimicrobial activity (Edwards et al., 2012). Furthermore, there is evidence that the neonate itself possesses several specific adaptations of its innate immune system. First, neonatal marsupials appear to possess an unusual number of cells associated with the innate immune system, in particular, neutrophils (Smith & Keyte, 2020). Additionally, a large variety of antimicrobial peptides (AMPs), for instance, cathelicidin antimicrobial peptides, are highly expressed in marsupial neonates, providing essential immune protection against pathogens (Borthwick et al., 2014; Park et al., 2024). Marsupials exhibit remarkable regenerative abilities, especially during the neonatal stage. The skin is capable of scarless healing, a trait that is not commonly found in adult mammals. Fetal wounds in marsupials heal without scarring due to the special composition (type III collagen) of their extracellular matrix that allows for better tissue regeneration and remodeling, and a low inflammatory response (Armstrong & Ferguson, 1995; Larson et al., 2010; Xia et al., 2018). Transplantation of allogeneic or xenogeneic skin grafts in eutherian and marsupial mammals can evoke strong immune responses that lead to acute rejection of the graft tissues (Zhou et al., 2013). Although adult Virginia opossums (*D. virginiana*) were shown to exhibit first and second set rejection phenomena to skin allografts, pouch young less than 12 days old did not reject such grafts. Graft survival in such young extended to at least 80 days in most cases (LaPlante et al., 1969; Old & Deane, 2000).

## 5 CONCLUSION

In summary, cutaneous respiration, characterized by a subepidermal capillary network with low diffusion distances, is an important adaptation in newborn marsupials and ensures the survival of the developing young in their initial days. It contributes to gas exchange before the lungs are fully functional. The early postnatal maturation of the cardiac system (closing of shunts by day 4) forces an early transition from cutaneous to pulmonary respiration. After ceasing cutaneous gas exchange, dermal growth and the formation of hair follicles and sweat glands take place. During postnatal development, the skin of the gray short‐tailed opossum undergoes massive structural changes, resulting in a functional shift from cutaneous respiration towards protection and insulation (thermoregulation).

## CONFLICT OF INTEREST STATEMENT

None declared.

## Supporting information


**Table S1.** List of Gray Short‐tailed opossum (*Monodelphis domestica*) specimens examined in this study.

## Data Availability

The data that support the findings of this study are made publicly available with Figshare. The original images are published under http://doi.org/10.6084/m9.figshare.27931593, and the data are published under http://doi.org/10.6084/m9.figshare.27931692.
