# Supplementary material for: Skin development in the gray short‐tailed opossum (Monodelphis domestica)—From skin respiration to thermoregulation
Source: J Anat. 2025 Feb 20;247(1):108–33. doi: 10.1111/joa.14236 (PMC12159325; doi:10.1111/joa.14236)
Supplement: Supplementary file 1 — Table S1. List of Gray Short‐tailed opossum (Monodelphis domestica) specimens examined in this study. [file JOA-247-108-s001.docx]

Supplement table: List of Gray Short-tailed opossum (*Monodelphis domestica*) specimens examined in this study

| Age (remark) | No. | Body weight (g) | Section | Staining | Method | |
| --- | --- | --- | --- | --- | --- | --- |
| 13 dpc  0 dpn | 2095e  2095g  1965_5  1965_6  1965_1  337 | -  -  0.11  0.11  0.12  0.11 | 5 µm, transversal  3 µm, transversal  8 µm, longitudinal  8 µm, transversal  1 µm, transversal  10 µm, transversal | H&E  Toluidin Blue  H&E  H&E  Toludin blue  Azan | LM  LM  LM  LM  LM  LM | |
| 2 dpn | 2383_1 | 0.14 | 6 µm, transversal | Movat | LM | |
|  | 2383_2 | 0.12 | 6 µm, transversal | Movat | LM | |
|  | 2383_3 | 0.13 | 6 µm, transversal | Movat | LM | |
| 4 dpn | 1825_1 | 0.20 | 8 µm, transversal | H&E | LM | |
|  | 3003A | - | 8 µm, transversal | H&E | LM | |
|  | 1995_2 | 0.21 | 1 µm, transversal | Meth./Azur | LM | |
| 7 dpn | 3006A | 0.34 | 8 µm, transversal | H&E | LM | |
|  | 2383_2 | 0.28 | 6 µm, transversal | Movat | LM | |
|  | 2383_4 | 0.27 | 1 µm, transversal | Meth./Azur | LM | |
| 11 dpn | 2419_1 | 0.47 | 6 µm, transversal | Azan | LM | |
|  | 2419_2 | 0.36 | 6 µm, transversal | Toludin blue | LM | |
| 14 dpn | 3014A | 1.00 | 8 µm, transversal | H&E | LM | |
|  | 1994_8 | 0.99 | 6 µm, transversal | Toludin blue | | LM |
|  | 1994_9 | 0.98 | 1 µm, transversal | Meth./Azur | | LM |
| 21 dpn | 2038 | 2.46 | 6 µm, transversal | Azan | | LM |
|  | 2039 | 2.39 | 6 µm, transversal | H&E | | LM |
| 28 dpn | 2041 | 4.78 | 6 µm, transversal | Azan | | LM |
|  | 2042 | 3.92 | 6 µm, transversal | Azan | | LM |
|  | 2043 | 5.15 | 6 µm, transversal | Azan | | LM |
| 35 dpn | 2061 | 7.70 | 6 µm, transversal | Azan | LM | |
|  | 2062 | 6.63 | 6 µm, transversal | H&E | LM | |
|  | 2065 | 7.25 | 6 µm, transversal | Azan | LM | |
| 49 dpn | 2045 | 12.05 | 6 µm, transversal | Azan | LM | |
|  | 2046 | 13.49 | 6 µm, transversal | H&E | LM | |
|  | 2402 | 11.59 | 6 µm, transversal | H&E | LM | |
| 57 dpn | 1642 |  | 6 µm, transversal | Trichrome | LM | |
|  | 2416 | 18.53 | 6 µm, transversal | H&E | LM | |
| Adult | 1986 | - | 6 µm, transversal | Azan | LM | |
|  | 2381 | 86.00 | 6 µm, transversal | H&E | LM | |
|  | 2383 | 65.50 | 6 µm, transversal | H&E | LM | |
| dpn. days post natum; H&E, Hämatoxylin & Eosin; Meth./Azur, Methylenblau / Azur II | | | | | | |
